# Functionally complementary bacterial inoculant coordinates arbuscular mycorrhizal fungi to improve *Angelica sinensis* root yield and quality

**DOI:** 10.1002/imo2.70091

**Published:** 2026-03-14

**Authors:** Zongyu Zhang, Yun Wang, Jiecai Zhao, Gaofeng Chen, Andéole Niyongabo Turatsinze, Liang Yue, Yuan Tian, Xiaofan Xie, Ailing Ye, Meilan Zhang, Lingling Wu, Zengxiang Guo, Yubao Zhang, Ruoyu Wang

**Affiliations:** ^1^ State Key Laboratory of Ecological Safety and Sustainable Development in Arid Lands, Northwest Institute of Eco‐Environment and Resources Chinese Academy of Sciences Lanzhou China; ^2^ Lanzhou Eco‐Agriculture Experimental Research Station, Northwest Institute of Eco‐Environment and Resources Chinese Academy of Sciences Lanzhou China; ^3^ Key Laboratory of Desert and Desertification, Northwest Institute of Eco‐Environment and Resources Chinese Academy of Sciences Lanzhou China; ^4^ Gansu Shangnong Biotechnology Co. Ltd Baiyin China; ^5^ University of Chinese Academy of Sciences Beijing China; ^6^ Gansu Minxian Angelica Research Institute Dingxi China

**Keywords:** AMF, *Bacillus*, composite microbial agent, medicinal yield and quality, PGPR, *Pseudomonas*, Radix *Angelica sinensis*

## Abstract

Radix *Angelica sinensis* (RAS) is a high‐value medicinal herb whose productivity and medicinal quality are often constrained by soil‐borne diseases and suboptimal rhizosphere conditions. Plant growth‐promoting rhizobacteria (PGPR) offer a sustainable alternative to chemical inputs, yet the efficacy of different microbial combinations and their functional mechanisms remains unclear. Here, we tested three PGPRs (one *Pseudomonas* isolate and two *Bacillus* strains) to *A. sinensis* rhizospheres. We showed that the three‐member composite microbial agent (CMA) exhibited the most pronounced effects on promoting growth, increasing yield, suppressing disease, and boosting the accumulation of bioactive compounds. We further observed that treatments containing *Bacillus*‐based inoculants enhanced the colonization of arbuscular mycorrhizal fungi (AMF) in RAS at harvest. These AMF likely utilize root‐secreted and rhizosphere‐enriched lipid compounds (palmitic acid, linoleic acid, and sphingolipids) both as carbon source and as symbiosis signals, thereby promoting root growth and helping protect against pathogens. Interestingly, a single *Pseudomonas*‐based formulation performed on par with the three‐strain consortium across multiple functional traits but showed limited effects on the accumulation of medicinal metabolites. This unique effect is likely linked to its capacity to enrich nitrogen in the rhizosphere soil, a change that is closely tied to fungal and nematode disease suppression. Furthermore, the CMA consortium improved the quality of RAS by enhancing its phenylpropanoid pathways. Taken together, our findings highlight functionally complementary microbial inoculants as an attractive and sustainable strategy to enhance the value of medicinal plants by simultaneously increasing yield and improving agronomic quality.

## Highlights

- The three‐strain composite microbial agent effectively harnessed its functional potential to enhance Radix *Angelica sinensis* growth, strengthen resistance to coarse bark disease, promote accumulation of bioactive medicinal compounds, and optimize rhizosphere conditions.
- The *Bacillus* members of the composite microbial agent facilitated arbuscular mycorrhizal fungi colonization in plant roots, thereby establishing a cross‐kingdom consortium that exerts synergistic benefits to the host.
- *Pseudomonas* contributes to strong disease suppression and growth promotion by enriching nitrogen and stabilizing rhizosphere networks, yet it shows limited capacity to elevate bioactive metabolite levels in Radix *Angelica sinensis*.
- This study presents an efficient multi‐strain microbial inoculant whose members act cooperatively, demonstrating broad potential across diverse root‐ and rhizome‐based medicinal plants to suppress soil‐borne diseases and improve overall yield and quality.

## Graphical abstract

Comprehensive understanding of how diverse PGPR strains enhance the rhizosphere microenvironment remains a considerable challenge. Here, we provide experimental evidence that a functionally synergistic composite microbial formulation can markedly enhance growth performance and improve the quality attributes in *Angelica sinensis*. Our findings present a practical strategy for simultaneously controlling soil‐borne diseases and boosting the medicinal value of root‐ and rhizome‐based medicinal plants.

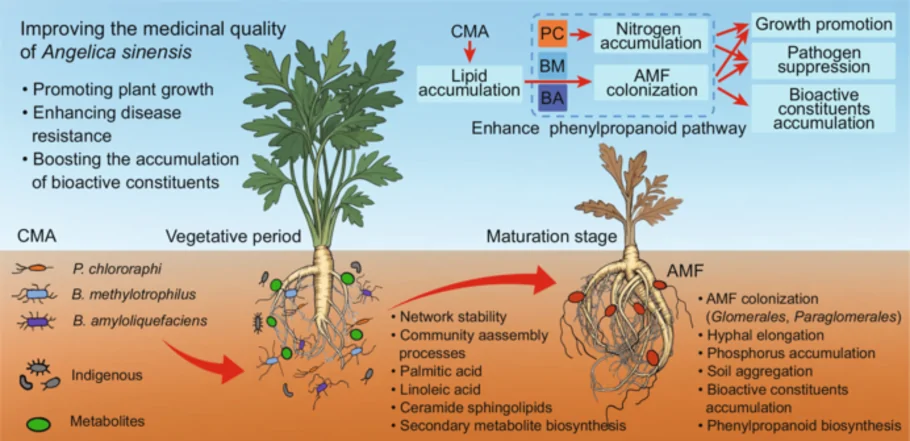

## 1 INTRODUCTION

Radix *Angelica sinensis* (RAS), also known as Chinese Angelica Root or Danggui, is the dried root of *Angelica sinensis* (Oliv.) Diels from the Apiaceae (Umbelliferae) family and represents a core herbal drug widely used in traditional Chinese medicine (TCM). Its distinct therapeutic features and extensive use in TCM clinical practice have made *A. sinensis* a long‐standing subject of interest for researchers in China and abroad. Benefiting from its robust pharmacological activities—most notably in tonifying Qi, stimulating hematopoiesis, modulating immune function, protecting the heart, regulating menstruation, and alleviating pain—RAS has been continuously incorporated into traditional medicinal preparations for more than 2000 years [1, 2]. The reputation of RAS as a premium Chinese herbal medicine is mainly attributed to its rich profile of bioactive components, including volatile oils, phenolic acids, phthalein and coumarin compounds, polysaccharides, and various flavonoids. Among these, ferulic acid and Z‐ligustilide serve as core compounds for its quality evaluation [3, 4]. The plant is predominantly cultivated in cold, moist, and high‐altitude (~1800 to 3300 m) regions of northwestern and southwestern China. Minxian County in southeastern Gansu Province is recognized as the most representative geo‐authentic (Daodi) production area, characterized by an average yearly temperature of 6.1°C, annual precipitation near 650 mm, dark loessial soils as the dominant soil type, and elevations ranging from 2040 to 3750 m [5]. At present, the cultivation area of *A. sinensis* in Minxian exceeds 300,000 mu, with an annual yield of more than 65,000 tons, accounting for roughly 80% of the total national output and about 90% of China′s exports (obtained from Minxian County government website) [6]. The annual output value of the local RAS industry has already surpassed 5 billion RMB. However, in recent years, continuous monocropping combined with improper and imbalanced fertilization has caused ongoing degradation of soil health in areas where *A. sinensis* is cultivated [7]. This decline is characterized by increasingly severe continuous‐cropping barriers and a clear escalation in the occurrence and severity of soil‐borne diseases. Consequently, both yield per hectare and the medicinal quality of RAS have declined, severely limiting the sustainable development of the RAS industry. Notably, root rot and root coarse bark disease (commonly known as Makou disease or stem nematode disease, *Ditylenchus destructor* Thorne) have merged as the most destructive and prevalent disease in the major planting regions [8]. The etiology of coarse bark disease is still under debate, with evidence suggesting that both *Fusarium* spp. and nematodes may contribute to its manifestation [9]. Plant‐parasitic nematodes stand as a major agricultural pathogen, causing ~12.3% production losses and 157 billion economic impact per year globally [10]. In *Angelica* species and other high‐value herbs, infections by *Fusarium* spp. or nematode not only diminishes biomass but also critically degrades key bioactive constituents, directly compromising both therapeutic efficacy and market value.

Microbial communities inhabiting in plant roots and the surrounding rhizosphere soil are regarded as the plant's “second genome.” These microbes maintain intimate interactions with their host and, under natural conditions, participate in regulating plant growth and development while helping to maintain overall plant health [11, 12]. Plant growth‐promoting microbes (PGPMs) represent a promising, sustainable, and eco‐friendly strategy to address the inherent constraints of conventional agriculture [13, 14]. PGPMs establish beneficial symbioses with host plants, where they not only enhance nutrient uptake and stimulate hormone production, but also suppress rhizosphere pathogens through antibiotic synthesis and triggering host systemic immune resistance [15, 16]. In exchange, plants deliver energy‐rich compounds via root exudation and generate a microhabitat conducive to PGPM colonization. In addition to such targeted symbioses, host plants also actively shape broader rhizosphere microbial communities through the release of diverse metabolites, thereby recruiting additional beneficial taxa and promoting the long‐term stability of soil microbial networks [17]. Diverse beneficial bacterial strains have been widely applied as bioinoculants to colonize the rhizospheres of crops, vegetables, and medicinal plant species, such as *Bacillus velezensis* SQR9, *Bacillus amyloliquefaciens* W19, *Bacillus velezensis* FZB42, *Pseudomonas azotoformans* FAP5, *Stenotrophomonas maltophilia* SBP‐9 [7, 18, 19, 20, 21, 22]. It is generally accepted that, compared to inoculant composed of a single microbe, composite microbial agent (CMA) typically exhibits enhanced tolerance to environmental fluctuations, more diverse functional capacities, and higher community stability, largely driven by functional complementarity and cooperation among strains [23, 24, 25]. Generally, *Bacillus* spp. and member of the genus *Pseudomonas* are well‐studied beneficial taxa, with several strains already commercially applied in modern agriculture through seed inoculation, foliar spraying, and microbial bioformulations [18, 26]. Using solid carriers in the formulation of bacterial inoculants is an effective strategy to enhance microbial viability [27, 28]. Attapulgite clay (Mg_5_(H_2_O)_4_(Si_4_O_10_)_2_(OH)_2_·4(H_2_O)), a naturally abundant and cost‐effective layered silicate mineral with a large adsorption surface area and specialized microporous structure, provides an excellent platform for the sustained release of PGPRs. Granular bacterial inoculant (GBI) made of the clay have been broadly applicated in diverse agriculture systems, including improving soil structure, regulating soil acidity, adsorbing heavy metal ions, and serving as a slow‐release fertilizer, plant growth regulator, and pesticide carrier [20, 29]. Arbuscular mycorrhizal fungi (AMF) have co‐evolved with many terrestrial plants for over 400 million years, making them the most widespread mutualistic symbionts [30]. By colonizing plant roots, they protect the host against biotic and abiotic stress, and markedly enhance its uptake of diffusion‐limited nutrients, usually phosphorus (more than 90%) and nitrogen (more than 50%) [31]. In return, plant deliver photosynthetically fixed carbon to the colonizing fungi [32, 33]. The thin hyphae, as well as an extensively branched hyphal network allows AMF to access soil cavities for an increased root absorptive surface area. This plant‐AMF‐bacterium cross‐kingdom continuum in agriculture systems is gaining recognition as one of the key driving factors in suppressing soil‐borne pathogens, promoting production, and maintaining agricultural ecosystem stability [30]. However, the understanding of how the added PGPR alters the network of interactions within the indigenous bacterial and fungal communities and how it forms direct or indirect relationships with the host plant is still limited. This knowledge gap is particularly pronounced given the scarcity of reports on the application of composite microbial agents to key authentic medicinal herbs.

In this work, we aimed to identify the most effective microbial inoculant combination for significantly improving the medicinal quality of RAS and to investigate in depth its interaction mechanisms with the host plant. *Bacillus* and *Pseudomonas* species are well known for their strong capacity to stimulate plant growth, enhance abiotic stress resistance, suppress diseases, and improve the soil microenvironment [34, 35, 36, 37]. Earlier work has shown that when they engage with the host, they frequently trigger major metabolic routes in plants, such as lipid metabolism and phenylpropanoid‐related metabolism [38, 39]. Moreover, the mechanisms underlying their interactions with hosts exhibit a high degree of conservation across diverse crop species and ecosystems, offering a reliable framework for the development and application of microbial products [18, 26]. Accordingly, we selected three representative probiotic strains, covering the two bacterial groups of interest, from a collection of well‐performing biocontrol strains established in our previous work [40, 41, 42]. These strains include *P. chlororaphi* (PC), *B. methylotrophilus* (BM), and *B. amyloliquefaciens* (BA). Notably, strain BA has consistently demonstrated as a reliable biocontrol with plant growth‐promoting effects in a range of our prior studied plants, including rice (*Oryza sativa* L.), maize (*Zea mays* L.), potato (*Solanum tuberosum* L.), Lanzhou Lily (*Lilium davidii* var. *unicolor*), and Danggui (*A. sinensis*). Based on the different combinations of these three bacterial agents, we integrated phenotypic data with microbiome and metabolome profiles to systematically assess the individual and synergistic effects of each strain. Our results demonstrated that CMA (composite microbial agent), an optimal composite inoculant composed of all three members, significantly boosts the symbiotic colonization of AM fungi on the host root interface, a crucial factor underlying the enhanced medicinal quality and biomass production of RAS. Thus, our findings suggest a functionally synergistic microbial consortium that could provide novel perspectives for biocontrol of diseases in *A. sinensis* and other plants.

## 2 RESULTS

### 2.1 Field applications of GBI enhance *A. sinensis* growth

In our previous work, we obtained multiple candidate probiotic isolates with promising application value (Table S1). By integrating evidence from plant growth‐promoting traits, biocontrol performance, and genomic background, we selected three representative strains, PC (from potato tuber tissue), BM (from *A. sinensis* rhizosphere), and BA (from seabed silt), for a systematic assessment of their ability to enhance the growth and disease resistance in *A. sinensis* (Figure 1A and Figure S1). Interaction assays on Luria‐Bertani (LB) plates demonstrated that the colonies of all three isolates expanded radially in a uniform and symmetric pattern without any mutual inhibition in all tested combinations (Figure 1B), suggesting that their functional stability will be maintained in a composite microbial community. These tested strains were compounded with attapulgite mineral soil to produce GBI, which was evaluated in field trails at *A. sinensis* genuine producing areas in Minxian (Figure 1A,C). The application of bacterial inoculant resulted in positive and continuous improvemeny in plant growth almost throughout the growing period (Figure 1D–F). In July, all microbial inoculant treatments significantly promoted plant height. However, in the following 2 months, the effects diverged across treatments, with BM maintaining a significant promotion of height and others showing no statistically significant change. Compared to the control groups (CK), inoculant treatment significantly increased crown diameter and stem diameter in September. By harvesting, both PC and CMA treatment significantly enhanced plant root development, such as main root length, root fresh weight, and root dry weight (Figure 1G–K). Notably, the CMA treatment increased main root length and main root diameter by 17.9% (*p* < 0.001) and 21.3% (*p* < 0.01), respectively. The average root weight also showed an increase of 77.0% and 79.1% (*p* < 0.01) in PC and CMA groups, respectively. These microbial agents further exhibited substantial potential for enhancing the yield of *A. sinensis* medicinal materials, with significant yield improvement of 55.2%, 60.3%, and 52.2% (*p* < 0.001) in PC, PC + BM, and CMA groups, respectively (Table S2). Besides, our findings indicate that the use of GBI not only increased the proportion of first‐ and second‐grade medicinal herbs (especially in PC and CMA group) but also inhibited the development of *A. sinensis* coarse bark disease, with 67.1% (*p* < 0.01) and 49.9% (*p* < 0.05) reduction of moderate and severe disease incidence in PC and CMA group, respectively (Table S2). Finally, microbial agents also led to the accumulation of the primary medicinal compounds, among which the CMA group was the most effective, enhancing the level of ferulic acid by 53.6% and that of ligustilide by 81.6% (*p* < 0.001), respectively.

**Figure 1:**
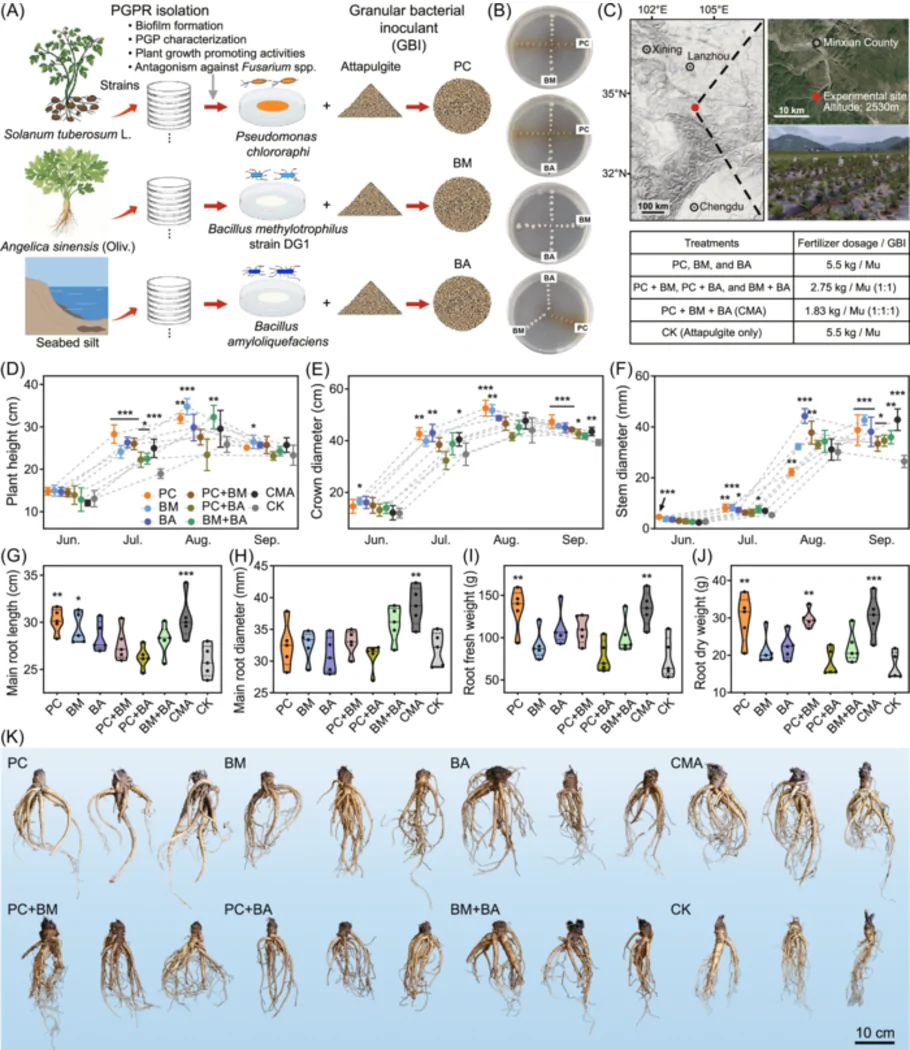
Formulation of bioinoculants and assessment of single and multiple consortia effects on the growth and morphology of *A. sinensis*. (A) Three high‐performing PGPR strains from distinct origins formulated with attapulgite clay to produce granular bacterial inoculants. (B) The interactions of two and three colonies in X‐ and Y‐shape on LB plate. (C) Geographical location and environmental characterization of study site, and experimental layout with different treatments. Line charts illustrating changes in plant above‐ground traits across different growth stages, including plant height (D), crown diameter (E), and stem diameter (F). Data are presented as mean ± SD. Asterisks indicate significant differences from the control (*p < 0.05, **p < 0.01, and ***p < 0.001). Violin plots showing the varied distributions of below‐ground root characteristics among different treatments at harvest, including main root length (G), main root diameter (H), root fresh weight (I), and root dry weight (J). Bars in the violin plot represent the median (solid line) and interquartile range (dotted line). (K) Root morphological traits at harvest. BA, *Bacillus amyloliquefaciens*; BM, *Bacillus methylotrophilus*; CK, control group; CMA, composite microbial agent; PC, *Pseudomonas chlororaphi*; PGPR, plant growth‐promoting rhizobacteria; SD, standard deviation.

### 2.2 Probiotic agents drive the alteration of microbial communities in *A. sinensis* root and rhizosphere soil

During the crucial development period of August, *A. sinensis* completes its emergence and slows the above‐ground growth, consequently focusing on the rapid establishment of the below‐ground root system. To investigate the effects of probiotic strains on indigenous bacterial and fungal communities in the *A. sinensis* root and rhizosphere, the microbial diversity and function characteristics were analyzed via high‐throughput 16S rRNA and ITS gene amplicon sequencing. Dilution curves indicated that sufficient amplicon sequence variants (ASVs) for bacteria and fungi analysis had been obtained (Figure S2). Alpha diversity analysis indicated that the addition of bioinoculants led to a significant decrease in root‐associated bacterial and fungal diversity (observed species, phylogenetic diversity, Chao1, Shannon, and Richness) in RAS compared to the control (Figure 2A,B and Figure S3). While the overall microbial diversity in the rhizosphere soil remained largely unchanged. Across all groups, microbiomes in soil maintained consistently higher diversity than that in root. These results indicate that the inoculants could not disturb the rhizosphere soil community, which is characterized by higher functional redundancy, remained relatively stable. However, dominant strains that successfully colonized the roots likely gain priority access to host root exudates, making the root‐associated community structure more prone to shifts. By nonmetric multidimensional scaling (NMDS) ordination of microbial communities, we further demonstrated that bacterial (16S rRNA) assemblages were strongly structured by habitat, with root and soil samples forming distinct clusters (Figure 2C). In contrast, fungal (ITS) communities did not exhibit clear separation, suggesting that fungal taxa may exhibit broader ecological tolerance or stronger dispersal between compartments, resulting in less habitat‐specific structuring. This pattern was consistently observed in principal coordinates analysis (PCoA), where fungal communities showed minimal separation among different experimental groups (Figure S4). Moreover, analysis of beta nearest taxon index (βNTI) revealed that the probiotic treatment selectively drove the assembly processes in rhizosphere soil of 16S rRNA sequence‐based microbiota (βNTI < −2), whereas the other communities were largely governed by stochasticity forces (Figure 2D). Subsequent investigations also showed that random events, such as dispersal limitation and ecological drift, outweighed deterministic environmental filtering in structuring microbial communities across these habitats (Figure S5). Interestingly, the soil bacteria exhibited a higher proportion of homogeneous selection compared to soil fungi and root microbiome, suggesting that the bioinoculant treatment did not impose a strong directional shift on soil environmental parameters, but maintained a degree of environmental stability that promoted convergence of specific microbial groups.

**Figure 2:**
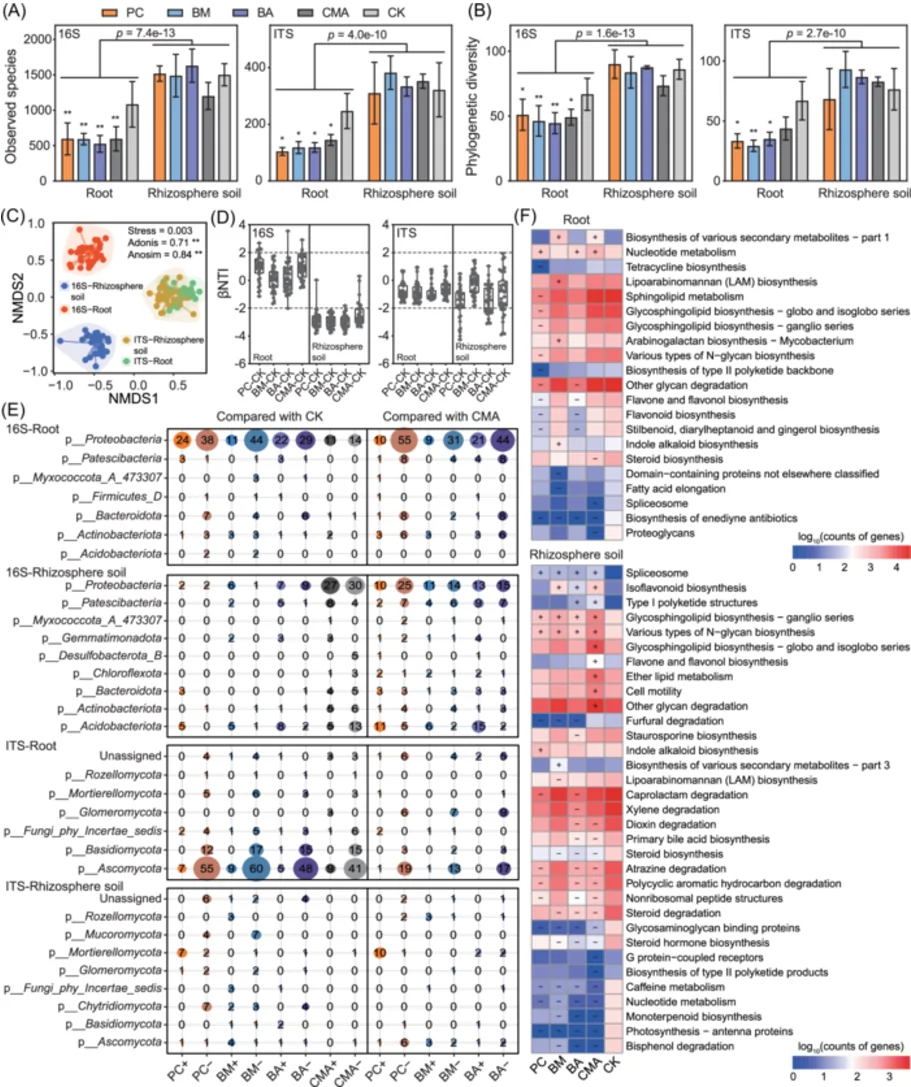
Bacterial and fungal diversity and functional potential under different inoculant treatments. Alpha diversity indices, including observed species (A) and phylogenetic diversity (B), across distinct compartments and groups. Data are presented as mean ± SD. Asterisks indicate significant differences from the control (*p < 0.05, **p < 0.01, and ***p < 0.001). (C) The beta diversity of root and rhizosphere microbiome community composition based on non‐metric multidimensional scaling (NMDS). Permutational multivariate analysis of variance (PERMANOVA) and analysis of similarities (Anosim) were conducted using treatments within each niche and microbial type as the grouping factors. Asterisks indicate significant differences (**p < 0.01). (D) Boxplot showing the distribution of beta nearest taxon index (βNTI) values for four treatment‐control groups from distinct niches. Horizontal dashed gray lines mark the upper (βNTI > 2) and lower (βNTI < −2) significance thresholds for determining the assembly pattern. (E) Bubble diagram depicting the number of phylum‐level ASVs showing statistically significant differences between treatment groups and control/CMA (*p* < 0.05 and log2fold change > 2). Bubble size and numeric labels denote the number of ASVs increased (+) or decreased (−). (F) Functional pathways predicted from significantly different ASVs of root and rhizosphere soil using PICRUSt2 (*p* < 0.05 and log2fold change > 1). Plus signs (+) in the heatmap indicate the pathways enriched in the treatment groups, while minus signs (−) denote pathways with significant enrichment in the control group. ASVs, amplicon sequence variants.

Microbial taxonomic compositions of *A. sinensis* root and rhizosphere soil samples from the five groups are summarized in Figure S6. In the endophytic bacterial community of root tissues, over 80% of the microbial taxonomic units were composed of *Proteobacteria*, *Actinobacteriota*, and *Bacteroidota*, while the dominant phyla of endophytic fungal community were *Ascomycota*, *Basidiomycota*, *Fungi_phy_Incertae_sedis*, and *Mortierellomycota*. Across all rhizosphere soil groups, *Proteobacteria*, *Acidobacteriota*, *Actinobacteriota*, *Gemmatimonadota*, *Chloroflexota*, and *Bacteroidota* dominated the microbial communities. The fungal *Mortierellomycota* and *Ascomycota* constituted up to 60% of relative abundance in soil samples. Differential abundance analysis using DESeq. 2 revealed distinct characteristics following bioinoculant application (Figure 2E and Table S3). In the root endosphere, *Ascomycota*, *Basidiomycota*, *Mortierellomycota*, and *Bacterioidota* exhibited a substantial change toward depletion, while *Proteobacteria*, *Actinobacteria*, and *Patescribacteria* responded in a more balanced manner, with nearly similar number of ASVs increasing and decreasing when compared to CK. In contrast, soil communities showed relatively modest responses, with fewer phyla undergoing significant changes. Compared to CK, CMA group induced a weak change in root *Ascomycota* abundance compared to single agent treatment, but an amplified shift in *Proteobacteria*, *Acidobacteria*, and *Patescibacteria*. PICRUST‐based functional prediction analysis indicated that the mixed‐strain inoculant samples were enriched in pathways related to biosynthesis of various secondary metabolites and nucleotide metabolism in RAS rhizosphere, including flavone and flavonol biosynthesis, isoflavonoid biosynthesis, glycosphingolipid biosynthesis, type I polyketide structures, and cell motility in soil (Figure 2F). Consistently, members of the order *Xanthomonadales* were predominantly linked to specialized metabolism and signaling, bio‐membranes structure and stability functions, and chemotaxis and mobility functions (Figure S7). The order *Haliangiales* and *Micropepsales* were closely related to antibiotics biosynthesis. Co‐enrichment of these functional microorganisms enhances the overall microbial adaptation and competitive advantage within the rhizosphere, thereby facilitating the establishment and persistence of mutualistic associations with the host plant. Functional inference with FUNGuild for differential fungal ASVs revealed distinct trophic strategies in the root versus the rhizosphere soil (Figure S8). In roots, the shifted taxa were dominated by saprotrophs and mixed pathotroph‐saprotroph‐symbiotroph guilds. In rhizosphere soil, the major groups were symbiotrophs, pathotroph‐saprotrophs, and saprotroph‐symbiotrophs. In summary, compared to the control, functional reorganization under different treatments tended to favor decomposers and multi‐trophic taxa inside roots, but was dominated by the reassembly of symbiotic interaction‐associated guilds in the rhizosphere.

### 2.3 Network‐based co‐occurrence patterns reveal root and soil microbial interactions

To characterize the holistic microbial interaction pattern, all inoculation‐treatment samples (referred as GBI group) were pooled separately for root and rhizosphere soil niches to construct comprehensive co‐occurrence networks (Figure 3A). We found that the topology of the co‐occurrence networks differed between the root and soil compartments. The global network in root contained 127 nodes connected by 474 edges, with a moderately modular structure (0.38). The soil network exhibited greater complexity (211 nodes and 673 edges, with a modularity of 0.4), indicating that the soil microbial communities are more interconnected than those associated with roots. This inference is supported by the observation that removing nodes at any hierarchical level consistently resulted in elevated natural connectivity, a pattern further corroborated by significantly higher network cohesion values (*p* < 0.001; Figure 3B,C). Notably, the co‐occurrence network of five treatment groups displayed greater variation in their resilience patterns. Inoculant introduction enabled the root microbial network to preserve elevated natural connectivity and robustness under equivalent disturbances, whereas the soil network showed the greatest structural fragility. Of which, PC was the only treatment sustaining high stability and forming more redundant network in both niche habitats, potentially buffering functional loss under environmental stress. In both global networks, bacteria occupied a substantial proportion of the keystone positions (Figure 3A,D,E and Table S4). Connectors were predominantly affiliated with *Proteobacteria*, *Actinobacteriota*, and *Chloroflexota*, accounting for over 74% of such nodes, while module hubs were mainly assigned to *Proteobacteria*, *Ascomycota*, and *Rozellomycota*. Rhizosphere soil network contained the highest number of network hubs, suggesting stronger cross‐module linkages driven by bacteria taxa under this condition. These patterns indicate that bacteria, particularly members of *Proteobacteria*, play a central role in maintaining network structure and facilitating interactions across microbial communities.

**Figure 3:**
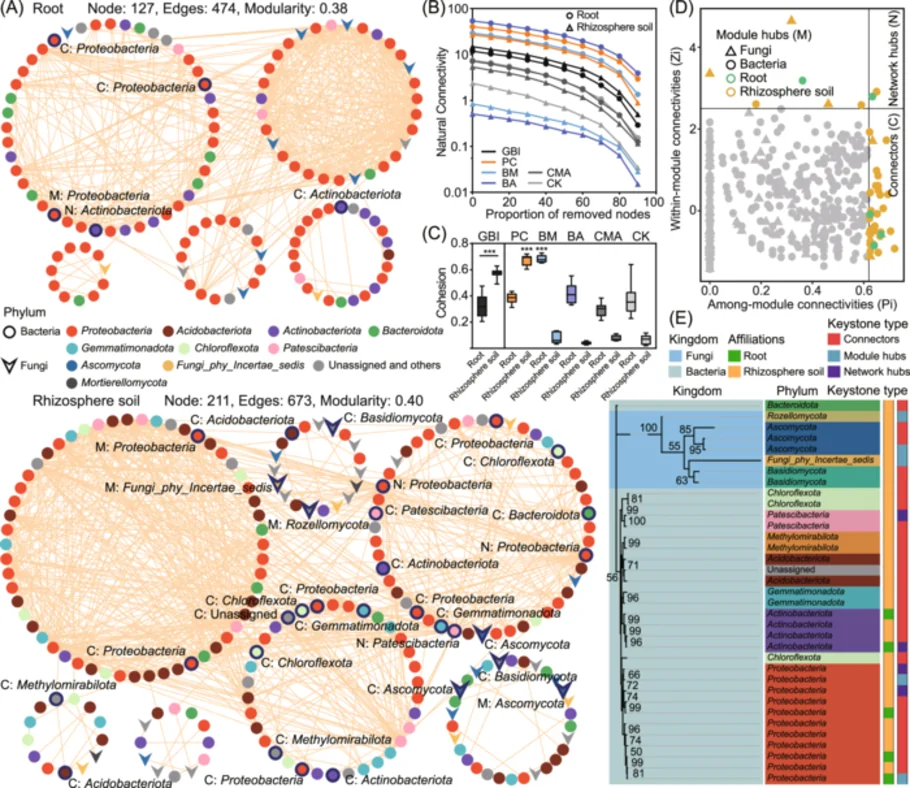
Co‐occurrence network of 16S and ITS amplicon sequence variants in the root and rhizosphere soil. (A) Module‐based association network of the global granular bacterial inoculant (GBI) samples in root and rhizosphere soil. Colors of nodes indicate the different major phyla. The taxonomy of keystone ASVs is labeled with dark blue circles. (B) Structural robustness of microbial network in different groups. (C) Network community cohesion. Asterisks indicate significant differences from the control (***p < 0.001). (D) Classification of nodes to identify putative keystone species within the global networks. (E) Phylogenetic tree of keystone ASVs in the networks. Bootstrap values higher than 50% are shown on the nodes.

### 2.4 CMA treatment promotes marked enrichment of lipid metabolites in RAS

To investigate the impact of microbial agent application on metabolite composition, we conducted an untargeted metabolomic analysis on *A. sinensis* roots and rhizosphere soil. In total, 4614 mass/retention on time features were detected in the negative electrospray ionization (ESI) and 4793 in positive modes across all samples. Orthogonal partial least squares discriminant analysis (OPLS‐DA) modeling confirmed a significant treatment effect, with clear separation between control and different treated groups for both sample types (root: *R* ^2^ *Y* = 0.974, *Q* ^2^ *Y* = 0.772; rhizosphere soil: *R* ^2^Y = 0.852, *Q* ^2^ *Y* = 0.673; Figure 4A). The robustness of these models was confirmed by high *R* ^2^ and *Q* ^2^ values, indicating a significant and reliable metabolic shift induced by the treatment (Figure S9). The variable importance in projection (VIP) score was calculated to assess the contribution of each metabolite to group separation, thereby allowing for their prioritization. To robustly identify key differential metabolites, we integrated two independent statistical methods: limma for differential abundance and OPLS‐DA‐derived VIP scores for discriminatory importance. This dual‐filter approach yielded a refined list of 32 top high‐abundance differential metabolites in the roots and 24 in the rhizosphere soil for CMA and CK samples (log_2_fold change > 2.5 with *p*‐adj < 0.001 and VIP > 1.8 with *p* < 0.05; Figure 4B). Among these key metabolites, lipid‐like compounds N‐hexadecanoyl‐sphinganine (Cer(d18:0/16:0)) and N‐tetradecanoyl‐sphinganine (Cer(d18:0/14:0), log_2_fold change = 2.44) showed a strong association with the assembly of rhizosphere soil microbial communities based on a Mantel test (Figure S10 and Table S5). And their relative abundance exhibited 7.8‐ and 5.5‐fold increase, respectively, compared with the control treatment. Inoculation triggered marked alterations in the metabolic landscapes of both roots and the soil compared to the control (Table S5). In roots, gene set variation analysis (GSVA) revealed a consistent enrichment of phosphatidylinositol signaling system and glycerolipid metabolism pathway across all treatment groups compared to the control (Figure S11). Furthermore, out of 996 metabolites were annotated in the LIPID metabolites and pathways strategy (LIPID MAPS) database, and 120 were linked to Fatty Acids and Conjugates (FA01). Differential analysis further revealed that FA classes of straight‐chain fatty acids and unsaturated fatty acids were predominantly upregulated in the treated roots (Figure 4C). Of which, CMA treatment caused palmitic acid and linoleic acid to increase by 2.36 and 7.20 times compared to CK, respectively.

**Figure 4:**
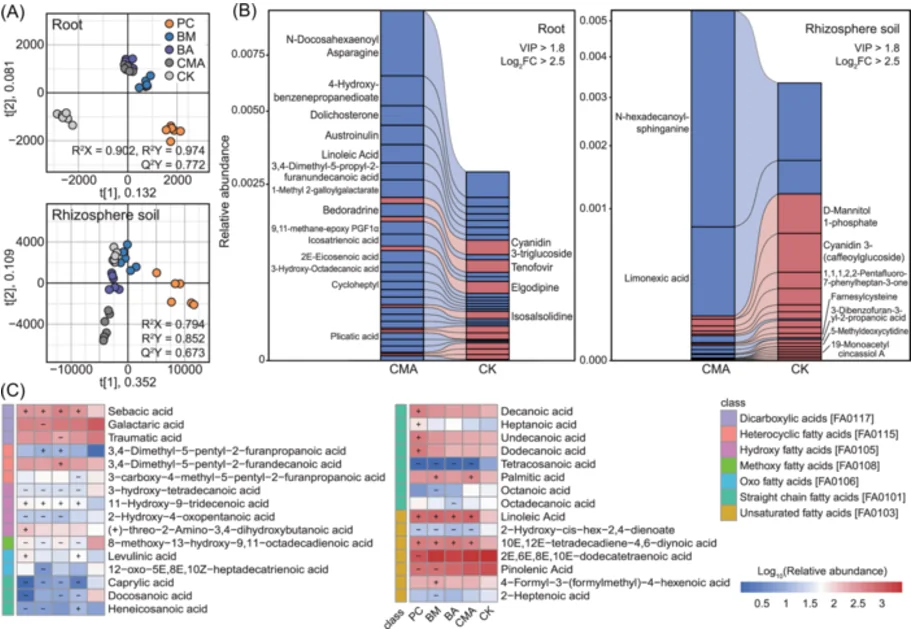
Metabolic profiling of *A. sinensis* root and rhizosphere soil. (A) OPLS‐DA statistical model of root and rhizosphere metabolites. (B) The flow plot showed the relative abundances of individual top‐ranked differential metabolites in CMA and CK (VIP > 1.8 and log_2_fold change > 2.5). Metabolites significantly enriched in CMA are highlighted in blue and those in the CK are colored red. (C) The heatmap displays the abundance of differential lipid metabolites in roots (*p* < 0.05, log_2_fold change > 1). The heatmap uses “+” to indicate metabolites showing significant enrichment in the treatment group, “−” to indicate those enriched in the control group. FC, fold change; OPLS‐DA, orthogonal partial least squares discriminant analysis; VIP, variable importance in projection.

### 2.5 *Bacillus*‐like PGPR promotes the colonization of AMF in host roots

It is known that AM fungi rely on fatty acids secreted from plant roots as a key carbon supply for their growth and development. To explore this possibility, we quantified the colonization of AMF in *A. sinensis* roots. Distinct hyphal structure was frequently observed on CMA root tissues using staining and microscopy examination (Figure 5A). CMA showed the highest AMF colonization rate (40.2%) and the longest mean hyphal length (132.5 μm), compared with 12.4% and 73.1 μm in CK (Figure 5B,C). The quantitative PCR further confirmed that AMF gene copy number in CMA was about 6.9 times that of the control, consistent with morphological observations (Figure 5D). Notably, *Bacillus*‐like inoculation markedly increased AMF colonization in RAS compared with the control. In contrast, *Pseudomonas* treatment resulted in only 9.6% colonization and 55.5 μm hyphal length, both below control levels, indicating inhibitory effect. Besides, the GBI treatment appeared to benefit both plant phosphorus nutrition acquisition and the surrounding soil environment (Figure 5E–G). In the CMA group, the combined action of PGPR and AMF significantly reduced the proportion of microaggregates (<0.25 mm) and medium macroaggregates (0.9–2 mm) in the rhizosphere soil (*p* < 0.05), and led to a trend of increase in the overall soil diameter (statistically non‐significant). A higher proportion of large macroaggregates strengthened soil structure, fostering conditions that favor nutrient preservation and persistent plant‐microbe associations. To further characterize the fungal community, we employed amplicon sequencing of the fungal ITS region (Figure 5H). Taxonomic profiling revealed that members of *Glomerales* and *Paraglomerales* dominated the fungal assemblage in the RAS of CMA (Figure 5I). Differential analysis confirmed their enrichment, suggesting that the introduced inoculant selectively influenced the composition of the resident AM fungal community (Figure 5J).

**Figure 5:**
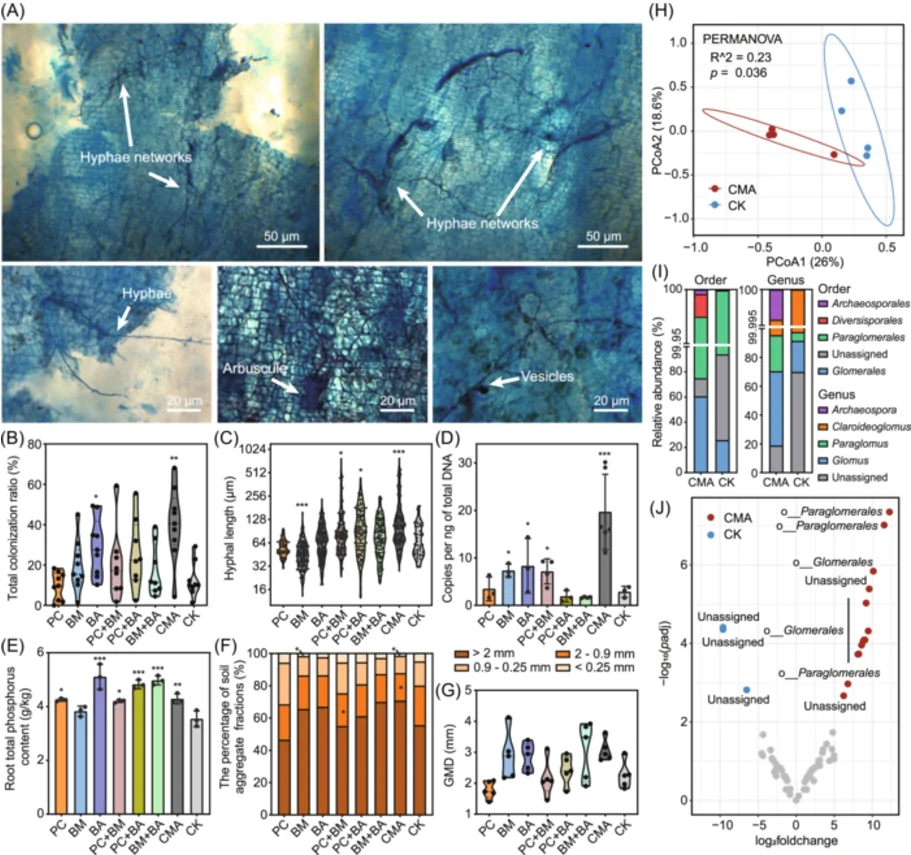
Qualitative and quantitative assessment of AMF colonization in RAS at harvest. (A) Microscopy images showing AMF colonization in CMA root tissues. (B and C) Variations in AMF total colonization ratio and hyphal length under different treatments. Bars in the violin plot represent the median (solid line) and interquartile range (dotted line). Asterisks indicate significant differences from the control (*p < 0.05, **p < 0.01, and ***p < 0.001). (D) AMF gene copy numbers in RAS estimated by qPCR. Data are presented as mean ± SD. (E) Changes of root total phosphorus content. (F) Impact of microbe‐host interactions on soil aggregate of rhizosphere soil. (G) Changes of soil aggregate stability index (geometric mean diameter, GMD). (H) Principal coordinate analysis (PCoA) and permutational multivariate analysis of variance (PERMANOVA) of AMF communities in CMA and CK. (I) Community composition and relative abundance of the 18S AMF gene amplicon sequencing at the order and genus levels. (J) Volcano plot depicting differential AMF ASV abundance between CMA and CK. Significantly enriched fungal ASVs in CMA and CK are shown in red and blue, respectively. AMF, arbuscular mycorrhizal fungi; RAS, Radix *Angelica sinensis*.

### 2.6 CMA treatment activates phenylpropanoid biosynthesis in RAS at harvest

Application of CMA led to superior outcomes in *A. sinensis* at harvest stage, including effective disease control, growth stimulation, accumulation of key bioactive constituents, and improvement of overall quality. To gain deeper insight into the biological processes, we conducted proteomics analysis and 16S rRNA amplicon sequencing of root endophytic bacteria. Compared to the control group, the relative abundance of *Proteobacteria* and *Bacteroidota* decreased, whereas *Actinobacteriota* and *Firmicutes_D* increased (Figure 6A). Functional prediction based on differential taxa indicated that microbes enriched in CMA treated roots were predominantly linked to Kyoto Encyclopedia of Genes and Genomes (KEGG) pathways associated with stress adaptation and defense responses, including phenazine biosynthesis, betalain biosynthesis, and nitrotoluene degradation (Figure 6B). These functional traits suggest that beneficial microbes may assist host in responding to both biotic and abiotic stress while contributing to pathogen suppression, thereby exerting a potential biocontrol effect. By contrast, the microbiota of control plants remained focused on the gradual breakdown of diverse metabolites, providing background energy supply. Besides, CMA treatment significantly increased root bacterial community diversity at harvest (Figure S12). KEGG pathway enrichment of the differentially expressed proteins further highlighted phenylpropanoid biosynthesis as one of the most significantly overrepresented pathways (Figure 6C). Network topology analysis of protein‐protein interactions (PPI) also placed this pathway in a central hub position, co‐localizing with ribosome, metabolic pathways, and biosynthesis of secondary metabolites modules (Figure 6D and Table S6). Mantel test results showed that endophytic bacteria abundance correlated strongly with ferulic acid and four PPI pathways, while AM fungi were more associated with ligustilide, AMF copies, as well as key protein pathways (Figure 6E). Notably, both active compounds exhibited significant positive correlations with the phenylpropanoid biosynthesis pathway, as well as with the copy number of the AMF gene. To further verify that PGPR can activate the plant phenylpropanoid pathway network, we co‐cultured *Bacillus amyloliquefaciens* (BA) with *Arabidopsis* and performed transcriptome sequencing of shoots and roots. Differential expression analysis identified 386 differentially expressed genes (DEGs) in shoots and 3521 DEGs in roots (log_2_fold change > 1 and *p*‐adj < 0.05). KEGG analysis revealed strong enrichment of the phenylpropanoid biosynthesis pathway in both tissues, indicating a coherent activation of phenylpropanoid‐associated program following PGPR inoculation (Figure S13). These findings suggest that beneficial microbial colonization, phenylpropanoid biosynthesis, and the accumulation of active medicinal compounds may operate in close coordination, forming a functional core that supports the overall enhancement of RAS medicinal quality.

**Figure 6:**
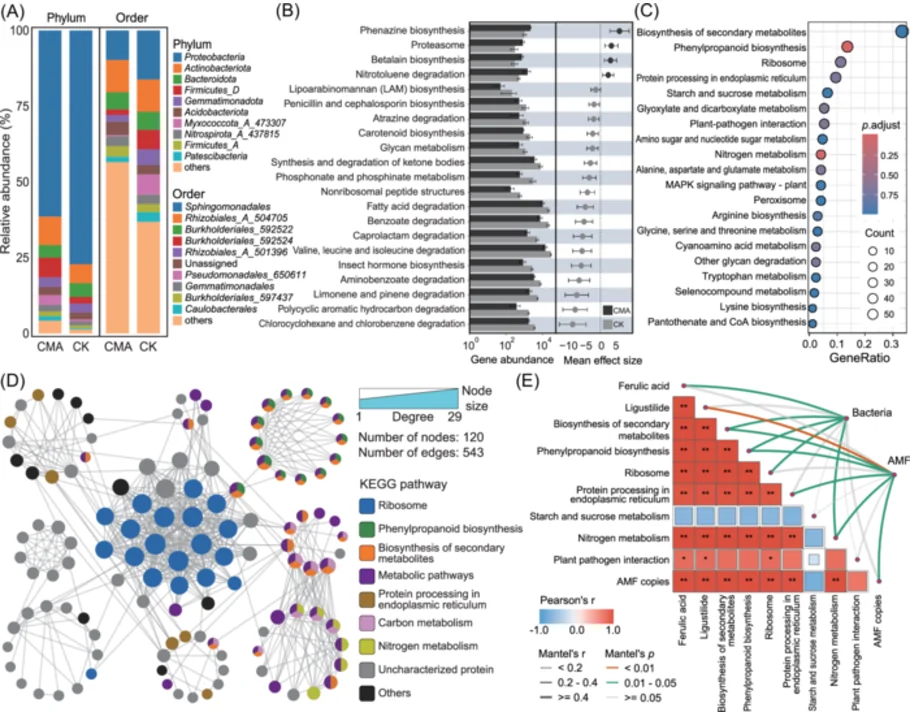
Modulation of microbiota–host interactions on critical biological pathways in the RAS of CMA at harvest. (A) Taxonomic composition of root‐associated endophytic bacterial communities at the phylum and order levels. (B) Functional potentials of bacterial communities in the root samples. Bar charts show mean value with standard deviation (SD) for each category. Error bars indicate standard errors (SE), and each point represents the percentage change in effect size of root bacterial community functional potential between CMA and CK, with 95% confidence intervals (CIs). Means below zero indicate higher functional potential in CK, whereas values above zero reflect greater potential in the treatment group. (C) Kyoto Encyclopedia of Genes and Genomes (KEGG) pathway enrichment analysis of differentially expressed proteins (DEPs, *p* < 0.05, log_2_fold change > 1.2) between CMA and CK. (D) Visualization of protein–protein interaction (PPI) network for the significantly DEPs. Colors of nodes indicate the different KEGG pathways. Only relatively abundant annotated pathways are shown for visualization. The complete KEGG pathway annotation for each protein is provided in Table S6. (E) Relationships between root‐associated endophytic bacteria and AMF of RAS and (i) levels of bioactive metabolites, (ii) key protein pathways, and (iii) AMF gene copy numbers.

### 2.7 The integrated profile of microbial inoculant effects on RAS quality

The structure equation model provided a clear picture of how varied applications of microbial agents shape aboveground biomass variation through their impacts on microbial community abundance and metabolite structure (Figure 7A). The five inter‐group treatments exerted a direct positive influence on the alpha‐diversity of root (standardized path coefficient = 0.61, *p* < 0.001) and rhizosphere soil (0.40, *p* < 0.05), and likewise, on the overall metabolomic composition within these compartments (0.33, *p* < 0.05 and 0.57, *p* < 0.01). The group differences directly and indirectly affected rhizosphere soil community structure and root metabolomics characteristics, which in turn weakly promoted aboveground biomass in August. AMF colonization may indirectly alter the accumulation of RAS bioactive compounds by directly affecting changes in hyphae length and the number of AMF copies (0.70, *p* < 0.001), as well as nutrient levels in rhizosphere soil (0.47, *p* < 0.05). The reduction of root NO_3_^−^‐N and the pronounced increase of rhizosphere soil NO_3_^−^‐N, compared to the control, were both associated with negative contributions to ferulic acid and ligustilide accumulation, with the estimated value of –1.16 and –0.81 (*p* < 0.001; Figure 7A and Figure S14). Conversely, a modest elevation in soil ammonium nitrogen seemed to facilitate RAS volatile oil accumulation (0.85, *p* < 0.001). Across all treatments, linear models revealed distinct patterns in the contribution of AMF colonization and nutrient levels to *A. sinensis* herb yields and bioactive constituents (Figure 7B). In nearly all treated groups, nutrient‐related characteristics contributed positively to herb production, in contrast to the control group (*p* < 0.05). Except for groups PC and BM + BA, indices related to AM fungi establishments in the root system were invariably associated with the accumulation of key bioactive compounds in the full model (*p* < 0.01).

**Figure 7:**
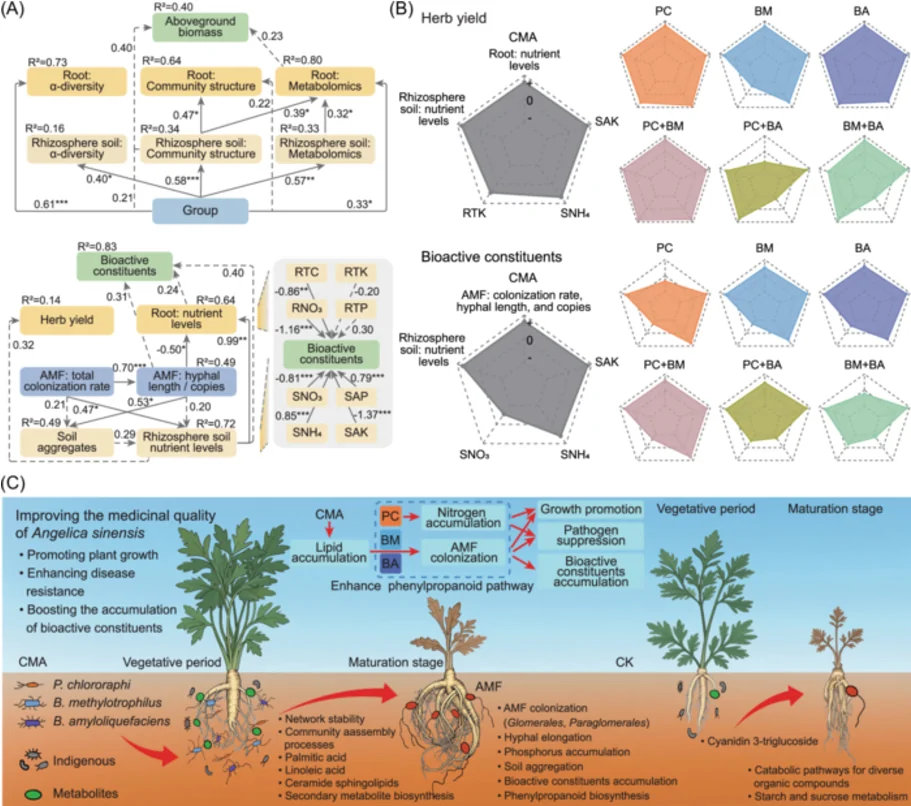
Overview of contribution analysis evaluating the effects of bio‐inoculant application, microbial community profiles, and changes in soil attributes on *A. sinensis* biomass production and product quality. (A) Structural equation modeling (SEM) of the upper panel showing the relative importance of bio‐inoculant treatments, microbial diversity, community structure (assembly processes and co‐network), and metabolite profiles on aboveground biomass production in *A. sinensis* at the vegetative period in August. The lower panel assessing how AM fungal colonization, soil structure, and nutrient availability influence the harvest‐stage yield and pharmacologically active constituents of RAS, through both direct and mediated effects. Solid grey lines denote statistically significant pathways (*p* < 0.05); dashed grey lines represent paths that are not statistically significant but have standardized path coefficients greater than 0.2 or less than −0.2. Numbers adjacent to lines show the standardized path coefficients, and asterisks denote significant paths (*p < 0.05, **p < 0.01, and ***p < 0.001). Model‐explained variance, expressed as *R*² values, is displayed alongside each response variable. (B) Radar charts illustrate the relative effects of different predictors, including root‐related traits, AMF characteristics, and nutrient profile metrics, on the core variable herb yield and bioactive constituents across bio‐inoculant treatment groups at harvest. Positive values (at the corners) indicate that the predictor exerts a stronger influence on variable in the treatment group compared to the control, whereas negative values (in the center) reflect a weaker or reversed influence. Only predictors with statistically significant changes (*p* < 0.05) are shown. (C) The conceptual model illustrates the functional complementarity among three beneficial strains within CMA, driving the restructuring of rhizosphere cross‐domain microbial assemblages, key metabolic compounds, and soil nutrients, ultimately improving both RAS yield and quality. RNO_3_, root NO_3_^−^N; RTC, root total carbon; RTK, root total potassium; RTP, root total phosphorus; SAK, soil available potassium; SAP, soil available phosphorus; SNH_4_, soil NH_4_^+^‐N; SNO_3_, soil NO_3_^—^N.

## 3 DISCUSSION

Plant‐associated beneficial microorganisms exhibit tremendous potential and promise for improving quality and productivity and enhancing stress resistance of diverse agricultural and medicinal plants [43, 44]. Current efforts to improve the rhizosphere micro‐environment of economically important plants are primarily focused on the composite microbial fertilizer or synthetic microbial community approaches [34, 45, 46, 47, 48]. In this study, we combined three newly developed microbial probiotic strains into a functionally complementary consortium, and used multi‐omics approaches to systematically evaluate their synergistic effects on the rhizosphere microbiome and host metabolic responses. Our results demonstrate that applying a high‐performing, functionally complementary composite microbial agent to *A. sinensis* can effectively suppress soil‐borne disease in medicinal plants, increase yield and active compounds content, and improve soil microenvironmental conditions (Figure 7C).

### 3.1 Bio‐inoculants modulate the *A. sinensis* rhizosphere microenvironment

Forming biofilm synergistically and stimulating beneficial bacteria enrichment in the rhizosphere are hallmark features of PGPRs [18, 49]. Our preliminary scanning electron microscopy observations indicate that *Bacillus amyloliquefaciens* strains (BA in the study) show strong colonization on host roots [50]. This colonization appears to rely largely on biofilm formation, enabling tight attachment to the root surface and long‐lasting residence. Accordingly, we have highlighted biofilm‐forming capacity in strain screening and evaluation as a straightforward way to improve their ability to colonize plant roots (Table S1) [40, 41, 42]. From an interaction perspective, *Bacillus* and *Pseudomonas* spp. seem more likely to form a mutually beneficial partnership than a relationship dominated by competitive exclusion. Previous studies have revealed a stable, mutually compatible interaction between the beneficial strains *Bacillus* and *Pseudomonas*. When their cells come into direct physical contact, *Pseudomonas* activates its type VI secretion system (T6SS), which can exclude or suppress other potential microbial competitors [18, 26, 47]. At the same time, *Bacillus* builds an extracellular matrix shield through secretion and matrix assembly, which reduces the ability of the T6SS to infiltrate and damage the colony. Moreover, T6SS exposure also induces sporulation of *Bacillus* stains. This cross‐species interaction has been reported across diverse environmental settings, suggesting that it may be governed by conserved mechanisms operating across ecological niches. Consistent with these findings, our plate‐based assays revealed no obvious inhibition zone, suggesting that the tested strains mainly interact through compatibility and cooperation (Figure 1B).

Interspecific interaction and niche availability are thought to be important determinants for the successful establishment proliferation of bio‐inoculant [51]. The slow‐release property of attapulgite clay enabled PGPRs to colonize RAS from the seedling stage and persistently occupy favorable ecological niches. In our formulated microbial consortium, *Pseudomonas* and *Bacillus* engage in a unique mutualistic partnership, collaboratively remodeling the rhizosphere microenvironment through division of metabolic labor [52]. By modulating root exudation patterns and releasing microbial quorum‐sensing signals and volatile organic compounds, these strains, in concert with the host plant, selectively recruit and enrich a wide community of probiotic microbes within rhizosphere networks [53, 54]. This process enhances community priority effects, enabling high‐frequency interacting taxa to maintain a dynamic balance between actively attracting plant‐beneficial microbes and suppressing potential pathogens [55]. Our study showed that microbial agents restructured the rhizosphere co‐occurrence networks (Figure 3). In the GBI‐associated networks of the root and soil, most inferred interactions were positive or positive‐correlation links, up to 87% and 60% respectively. Concurrently, the significant reduction in root microbial diversity implies that applying the microbial inoculant may reinforce the selection process, resulting in enhanced environmental filtering effect. Instead, it more likely reflects the selective enrichment of resident taxa that are better aligned with host‐inoculant interactions, thereby favoring the retention of functional traits linked to the biosynthesis of relevant metabolites and improvement of stress tolerance, as summarized in Figure 2F [56, 57]. Such functions may enhance the ecological advantage and colonization capacity of key beneficial members in roots and rhizosphere, including the hub probiotics identified in our co‐occurrence network (e.g., *Pseudomonadales*, *Rhizobiales*, and *Actinomycetales*) as well as beneficial fungi established in RAS (e.g., *Glomerales* and *Paraglomerales*). The resulting community shift may bias microbiome assembly toward a disease‐suppressive, health‐promoting configuration and, in turn, foster normal plant development [58, 59]. This mechanistic framework matches the phenotype observed in our study: enhanced growth coupled with reduced disease (Figure 1 and Table S2). In summary, our results suggest that applying the inoculant boosts plant health via synergistic mechanisms: direct root colonization by PGPR and indirect microbiome tuning by strengthening interactions among beneficial taxa.

### 3.2 AMF colonization in *A. sinensis* roots is enhanced by the interaction between *Bacillus* and the host

Lipid exchange is widely viewed as a key metabolic basis for initiating and maintaining the host–fungus partnership [30, 60]. During mycorrhizal symbiosis, 16:0 2‑monoacylglycerol is transported from the root to peri‐arbuscular membrane (PAM) interface for fungal uptake [61]. At the same time, large amounts of fatty acids are released, including lauric acid (C12:0), *Cis*‐palmitvaccenic acid (C16:1ω5), and triglyceride (TAG) (C44:2, C16:1–C16:1–C12:0) [62]. In agriculture ecosystems, AMF is commonly used as biological inoculation to boost the growth performance of host plants, resulting a significantly activation of fatty acid metabolic pathways and accumulation of fungi‐related lipids profile in plants, such as linolenic acid (C18:3), linoleic acids (C18:2), methyl oleate (C18:1), methyl linoleate (C18:2), and so on [63, 64, 65, 66]. Interaction between PGPRs and *A. sinensis* results in high levels of accumulation of palmitic acid (C16:0) and linoleic acid (C18:2) in RAS, suggesting that these fatty acids probably involved in the process of arbuscular mycorrhizal symbiosis (Figure 4). Palmitic acid can serve as a carbon source and metabolic energy for promoting AM fungi asymbiotic growth, and researchers further found that combining palmitate with myristate not only accelerated fungal growth but also led to a substantial change in fatty acid composition of triacylglycerol [67]. Indeed, beyond their role as central components of mycorrhizal symbiosis, lipids constitute the plant‐derived membranes surrounding arbuscules, provide carbon transferred from the host to arbuscular mycorrhizal fungi, represent the main storage form of carbon within AMF, and act as signaling molecules initiating fungal developmental programs [68]. In the rhizosphere soil under CMA, we detected significant enrichment of ceramide compounds, members of the class of sphingolipids (SPs, or glycosylceramides), with their abundance patterns closely matching the bacterial community assembly process (Figure 4 and Figure S10). Sphingolipids are critical molecular components in the plant‐AMF symbiosis, underpinning the formation, maturation, and long‐term integrity of the endosymbiotic compartment (PAM) [69]. Previous findings suggest that the application of AM fungal and beneficial bacterial inoculants can trigger a pronounced activation of sphingolipid‐mediated signaling networks [70, 71]. The composition and distribution of sphingolipids are tissue‐specific, with oil‐rich tissues showing a pronounced enrichment of ceramide and α‐hydroxylation, and a concurrent scarcity of glycosyl inositol phosphoceramides and glucosylceramides [72]. Consistent with this pattern, CMA treatment in *A. sinensis* prompted a significant buildup of ceramide sphingolipids within the rhizosphere zone. This observation is consistent with the extensive AM fungal colonization of its root system.

Although both AMF and PGPR have been successfully used to improve the soil microenvironment for host plants, research on how PGPR promotes the colonization of AMF remains scarce [73, 74]. Our experiments were originally designed around bacteria inoculants, and interestingly, we found that the exogenous microorganisms robustly drove the colonization of AMF mediated by direct or indirect interactions. The effect was most dramatic in the CMA group, which displayed greatest performance in colonization rates and hyphal lengths with the dominant microbial genus taxa identified as *Glomus*, *Paraglomus*, *Claroideoglomus*, and *Archaeospora* (Figure 5). Notably, we found that AMF exhibits selectivity colonization, as it was more inclined to engage in symbiotic relationship with bacterium genus of *Bacillus* than with genus of *Pseudomonas*. The vast hyphae networks of AMF connect host plant roots to soil beneficial microbial, forming a unique niche for the transfer of energy and nutrients, like C‐P exchange [75]. Fungal hyphae and their associated thin water films enhance spatial mixing and strengthen interspecific interactions within the microbial community [76]. In return, the diverse soil bacteria harboring surface of AMF hyphae potentially promote hyphal branching via the release of branched‐chain FAs [68]. There is evidence that the bacteria‐derived methyl branched‐chain fatty acids are thought to act as stimulants or nutrients for fungal hyphae germination and formation of secondary spores [77]. In a recent discovery, AMF hyphal exudate carboxylates recruit the colonization of *Pseudomonas fluorescens*, and the symbiotic relationship triggers the upregulate expression of N_2_O reductase gene *nosZ*, the abundance of which was positively correlated with hyphal length density [78]. The interaction between AMF and phosphate‐solubilizing bacteria (PSB) *Rahnella aquatilis* resulted in an increase Pi uptake and transport effective along the extraradical hyphae and further enhance C‐P exchange at the arbuscular interface between plant and AM fungus [79].

### 3.3 Beneficial bacteria and fungi enhance the quality of medicinal herbs

Anchored onto attapulgite clay particles, these probiotic strains gain a controlled‐release advantage in soil, facilitating persistent cooperation with both the host plants and its AM fungal allies. Sharing the same ecological niche as *Fusarium* spp., *Ceratobasidium* spp., and root‐lesion nematodes (*Pratylenchus* spp.) in agriculture system, AMF confers benefits to its host that extend far beyond improving phosphorus nutrition and plant growth to include directly competing with pathogens for space and resources and efficiently activating systemic resistance in the host [31]. Separately, studies have shown that AMF species from the genera *Funneliformis* and *Glomus* had a reductive effect on *Pratylenchus* population densities [80]. Mycorrhizal root exudates of tomato have been reported to play an important role in *Meloidogyne incognita* control by reducing nematode movement and penetration [81]. *Glomus* species, such as *G. versiforme*, *G. intraradices*, *G. mosseae*, *G. claroideum*, and *G. constrictum* have proven effective at bio‐controlling in medicinal herbs and crops *Fusarium*‐related diseases [82, 83]. We observed that following CMA treatment, the relative abundance of the fungal genus *Glomus* in *A. sinensis* roots increased to 51.5%, while only 21.6% in the control (Figure 5).

In addition to their growth‐promoting effects, PGPR play a key role in plant protection by helping suppress diseases and insect pests. Biocontrol bacteria *Pseudomonas* and *Bacillus* can significantly antagonism against the growth of either *Fusarium* or nematodes [41, 84, 85]. Chitinase enzyme, glucanases, and proteases purified from species of *Bacillus* and *Pseudomonas* play a pivotal role in weakening the fungal cell wall of many pathogens [86]. Among these PGPRs, *Bacillus amyloliquefaciens* is commonly used by itself or in conjunction with other beneficial strains, largely because they harbor nine dedicated gene clusters for synthesizing antimicrobial secondary metabolites, including cyclic lipopeptides (antifungal activity) and polyketides (antibacterial activity) [87]. These bacterial metabolites trigger pathways of induced systemic resistance to protect plants from pathogenic microbes, viruses, and nematodes [88]. In our experiments, groups of *Pseudomonas* strains exhibited great ability to suppress *Angelica* root coarse bark disease than the groups of *Bacillus* bacteria, resulting a disease incidence of 17%, while around 28% for *Bacillus* isolates (Table S2). The fact that this gram‐negative bacterium is both a potent biocontrol agent and an outstanding growth promoter, significantly enhancing nearly all *A. sinensis* phenotypes even in the absence of AMF colonization. This finding points to a clear divergence in the biocontrol strategies utilized by *Pseudomonas* and *Bacillus* groups. Our results indicate that *Pseudomonas* treatment enriched *A. sinensis* rhizosphere nitrogen, especially nitrate‐N, while concurrently reducing nitrate‐N in root tissues. For pathogenic fungi, the nitrogen that truly supports growth is the fraction they can directly access and assimilate within infected roots. Lower internal root nitrogen, therefore, suggests two complementary outcomes: the pathogen faces a limited supply of usable N after invasion, constraining its proliferation; meanwhile, the plant may preferentially divert N toward producing defense‐related compounds such as salicylic acid (SA) rather than storing it [89]. In addition, elevated rhizosphere nitrate‐N often points to strengthened nitrification, coupled with an expansion of bacteria involved in nitrogen turnover. This shift can reshape community structure and increase network complexity, creating ecological exclusion that limits fungal establishment [90]. Together, the inoculant appears to “keep nitrogen outside the root”, generating an N distribution pattern that is unfavorable for pathogen entry and ultimately reducing disease incidence [91, 92]. Furthermore, a nitrogen‐rich rhizosphere can promote lateral root formation and tissue expansion through signals such as amino acids and auxin, steering more host carbon toward growth and biomass building [93]. In contrast, sustaining AMF symbiosis demands continuous lipid transfer (such as palmitic acid) across the PAM to maintain arbuscules and the external mycelial network, which carries a substantial carbon cost [61, 62]. When nitrogen availability already pushes the plant toward rapid growth, the relative carbon allocation to rhizosphere cooperation (lipid provisioning to AMF) and to secondary pathways such as volatile oil production may be reduced. Consistent with this, in nutrient‐rich soil environment, such as those with high N and P, the growth and colonization of AMF become restricted, as the host plants may receive less benefit from AMF and allocate less carbon to AMF [94, 95]. By contrast, *Bacillus*‐based inoculants appear more likely to stimulate lipid accumulation in the host, thereby promoting AMF colonization. Enhanced AMF not only improve mineral acquisition and root homeostasis, but also drive carbon‐based secondary metabolism via signaling and metabolic reprogramming, leading to higher levels of key bioactive constituents (e.g., ferulic acid and Z‐ligustilide) as well as products of the phenylpropanoid pathway [96]. Given their high demand for carbon backbones and limited direct dependence on nitrogen, these pathways tend to yield more obvious boosts in oil‑linked bioactive components in plants experiencing relatively low nitrogen accumulation.

The use of chemical fertilizers and pesticides in cultivated *A. sinensis* has increased the incidence of soil‐borne diseases, leading to an obvious decline in quality. Our results show that the application of composite agents with three‐member microorganisms (CMA) can effectively improve the soil microenvironment and simultaneously enhance both the quality and yield of medicinal herbs, resulting in a beneficial outcome for both aspects. The tradition holds that the quality of *A. sinensis* is positively associated with the abundance of ferulic acid and Z‐ligustilide. Herein, application of the CMA microbial agent led to a substantial increase in active components in *A. sinensis* roots, with the elevated levels of over 50% and 80% compared to the attapulgite soil blank. Similarly, endophytic fungi treatment was found to promote the accumulation of Z‐ligustilide and ferulic acid contents [97]. Advancing the search for functionally versatile PGPMs specifically adapted to *A. sinensis*, together with the development of optimally balanced microbial consortia, is anticipated to yield superior approaches for controlling soil‐borne diseases in RAS and other key medicinal plants. These innovations will not only strengthen biological disease suppression but also promote the long‐term quality and productivity of medicinal plant resources.

## 4 CONCLUSION

With converging evidences from phenotypic observations and multi‐omics profiling, we demonstrated that PGPR‐driven improvement in RAS growth, disease resistance, and quality could result from the following coordinated mechanisms: (i) reshaping the root microbiome, (ii) strengthening of rhizosphere ecological networks, and (iii) reprogramming host metabolism. The three‐strain mixture produced the most pronounced combined effect, characterized by consortium‐specific lipid accumulation, enhanced AM fungal colonization, and activation of the phenylpropanoid pathway, which together produced higher levels of bioactive constituents. Notably, *Pseudomonas* was more closely linked to rhizosphere nitrogen enrichment and improved network stability, consistent with a primary role in sustaining plant growth and health. In contrast, treatments containing *Bacillus* aligned more strongly with lipid remodeling, AMF establishment, and quality‐related compounds shifts. The non‐redundant functions within the consortium offer a practical blueprint for developing multi‐strain bioinoculants to support the eco‐friendly disease management and enhance the quality of medicinal materials, and lay a theoretical foundation for a “bacteria‐fungi‐soil‐host” integrated, sustainable modulation strategy.

## 5 METHODS

### 5.1 Bacterial inoculants and field experiment design

Three plant promoting and biological control agents, *Pseudomonas chlororaphi* strain T8 (PC), a Gram‐negative bacteria, *Bacillus methylotrophilus strain* DG1 (BM), a Gram‐positive bacteria, and *Bacillus amyloliquefaciens* FZB42 (BA), a Gram‐positive bacteria, were isolated from potato tubers (*Solanum tuberosum* L.), rhizosphere of healthy *Angelica sinensis* (Oliv.) plants, and seabed silt in South China sea, respectively [40, 41]. These well‐established microbial strains were provided by the Northwest Institute of Eco‐Environment and Resources of the Chinese Academy of Sciences and had been deposited in the China General Microbiological Culture Collection Center (CGMCC) under accessions of No. 30005, 30006, and 11481, respectively. The antagonistic or synergistic effects between and among different strains were investigated using a series of interaction assays to assess their mutual relationships. Briefly, a 1 μL aliquot of each seed culture was evenly spotted on LB agar plate to form either an X‐shape or Y‐shape, and the plates were then sealed and incubated at 28°C for 3 to 5 days to capture images. Furthermore, to establish an effective and efficient fermentation system, small‐scale trials were conducted using the candidate strains. Single colonies of each strain were first grown in LB medium before being inoculated into a small fermenter with the conditions of 36°C, 180 rpm, pH range 6.5–7.5, for 16–24 h. The fermentation broth underwent instantaneous high‐temperature spray drying at 120°C, resulting in the bacterial powder, which was subsequently mixed with attapulgite clay using the disk granulation method. Containing at least 5.0 × 10^9^ CFU/g viable bacteria, the dried solid attapulgite‐coated biocontrol inoculant, with a diameter size of 3–5 mm, features a disintegration cycle last 2–4 weeks under natural conditions [7].

The field experiment was carried out in Sigou Town (34°17' N, 104°02' E), Minxian County, Gansu Province, China, with a mean annual temperature of 6 degrees, annual precipitation of 600 mm, an altitude of 2530 m, and a soil type of Chernozem, from April to October 2023 (Figure 1C). Study field with a history of more than 10 years of continuous *A. sinensis* monoculture. Before planting, the entire field was uniformly ploughed and leveled (pH was 6.0), followed by the allocation of plots for each treatment. All blocks were arranged according to a randomized block design. The experimental design included 40 blocks distributed across five replicates for each treatment. Each block was 1 m in width and 9 m in length, and contained four rows of plants, with a plant density of 30 *A. sinensis* individuals per row. Blocks were separated by a 0.6‐m gap. The trial sites comprised of eight treatments, including three separate applications of granular bacterial inoculant, PC, BM, and BA, three pairwise combinations (PC + BM, PC + BA, and BM + BA) in a 1:1 ratio, a full mixture of all three inoculants equally (PC + BM + BA, 1:1:1), as well as a control group (CK), which was the same attapulgite carrier granules alone, without any bacterial powder added. All solid inoculant of eight treatments were each applied at a dosage of 5.5 kg per mu through hole‐base sowing, where *A. sinensis* seedlings were also placed during the same process [7].

### 5.2 Field observations of plant phenotypes and sample collection

During the period from June to September, covering the plant's seeding stage to vegetative development phase, various phenotypic traits related to plant growth, including plant height, crown width, and stem diameter, were assessed monthly across all treatments. Within each block, six individual plants were randomly selected for trait measurements. Their values were averaged to represent one replicate, resulting in five replicates per treatment. Main root length, main root diameter, fresh root weight, dry root weight, and disease incidence were observed and recorded at harvest stage in the end of October. RAS coarse bark disease was quantified as the percentage of infected plants and classified based on the root infection area. Root with damaged area of 10%–30% were considered a mild disease, while those with lesion area of 30%–50% were classified as moderate incidence and greater than 50% were severe. Roots of *A. sinensis* were classified in the field by morphological size, with Grade I (>250 g), Grade II (>150 g), and Grade III (>100 g).

Plant root and rhizosphere soil samples were collected at two sampling times: during the vigorous growth stage in August and at harvest in October. For each treatment, three blocks were randomly picked within each treatment. Two sampling sites located far apart were randomly selected within each block, and three root tissue subsamples were collected at random from each site. Subsamples were combined at equal mass to produce one pooled sample, defined as one replicate. This design yielded six replicates per treatment (3 blocks × 2 sites per block) for omics‐based sequencing analyses. Corresponding rhizosphere soils were also sampled following the same strategy to ensure consistency. For rhizosphere soil collection, the roots were carefully excavated and then gently shaken by hand to detach non‐firmly attached bulk soil. The soil adhering within about 1 cm of the root surface was collected as rhizosphere soil. All plant materials and soil samples were immediately frozen in liquid nitrogen and then stored at –80°C for further experimentation.

### 5.3 RAS and soil nutrient characteristics

The collected soil samples were air‐dried and passed through a 2‐mm sieve to determine their chemical properties. All soil and root samples collected during the harvest period were assayed for C‐related attributes (soil organic carbon, SOC, and root total carbon, RTC), N‐related attributes (SNH_4_ and SNO_3_ for soil NH_4_^+^‐N and NO_3_^−^‐N, as well as RNH_4_ and RNO_3_ for root NH_4_^+^‐N and NO_3_^−^‐N), P‐related attributes (soil available phosphorus, SAP, and root total phosphorus, RTP), and K‐related attributes (soil available potassium, SAK, and root total potassium, RTK). The chemical properties were measured using standard protocols: carbon content was assayed by the potassium dichromate oxidation titration heating method, nitrogen content was measured by spectrophotometry, phosphorus level was determined by Mo–Sb colorimetric method, and potassium concentration was quantified via flame photometry and inductively coupled plasma‐optical emission spectrometry [98].

### 5.4 Amplicon sequencing and bioinformatics analysis

Total genomic DNA were extracted from root and soil samples (PC, BM, BA, CMA, and CK collected in August, as well as CMA and CK in October) using the TGuide S96 Magnetic DNA Kit (Tiangen Biotech (Beijing) Co., Ltd.) following the manufacturer's instructions. DNA concentration and purity were assessed using NanoDrop 2000 UV–Vis spectrophotometer (Thermo Scientific). The V3–V4 hypervariable regions of the bacterial 16S rRNA gene were amplified using the two sets of 33F/806R primer pairs (root, F: CADACTCCTACGGGAGGC, R: ATCCTGTTTGMTMCCCVCRC; soil, F: ACTCCTACGGGAGGCAGCA, R: GGACTACHVGGGTWTCTAAT). The fungal internal transcribed spacer region was amplified using two universal ITS1 primer pair (root, F: CTTGGTCATTTAGAGGAAGTAA, R: TGCGTTCTTCATCGATGC; soil, F: CTTGGTCATTTAGAGGAAGTAA, R: GCTGCGTTCTTCATCGATGC). PCR amplified products were separated on agarose gel, purified using the Omega DNA purification kit (Omega Inc.), and finally, a total of 132 amplicon libraries were paired‐end sequenced (2 × 250 bp) on the Illumina NovaSeq. 6000 platform.

Raw sequence reads were initially subjected to quality filtering using Trimmomatic (v0.39) tools for each sample, with parameters set to LEADING:20, TRAILING:20, SLIDINGWINDOW:4:20, and a minimum read length (MINLEN) of 200. The DADA2 program, on the QIIME2‐2023.5 platform, was employed to trimming, denoising, and identifying of ASVs [99]. Paired‐end read denoising was carried out with denoise‐paired command, using “‐‐p‐trim‐left‐f 0” and “‐‐p‐trim‐left‐r 0” (no trimming at the 5' ends) and “‐‐p‐trunc‐len‐f 230,” and “‐‐p‐trunc‐len‐r 230,” which standardized both forward and reverse reads to a length of 230 bp. Taxonomic annotations of representative ASVs were achieved based on the Naive Bayes classifier using Greengenes2 and UNITE database [100, 101]. Sequences identified as chloroplasts, mitochondria, or archaea were removed from the final ASV table. Rarefaction curves indicate the completeness of ASVs across the different treatments. ASV counts from all samples were standardized by rarefaction to the same sequencing depth in R package vegan (v2.6‐8). The q2‐diversity plugin in QIIME2 was used to conduct alpha diversity comparisons and rarefaction curves analysis. NMDS and PCoA analyses were performed to estimate and visualize beta diversity of samples with the R vegan package. Beta diversity was statistically analyzed using Permutational Multivariate Analysis of Variance (PERMANOVA) based on Bray–Curtis distances. Community assembly was evaluated under a null‐model approach using βNTI and beta net relatedness index (βNRI) with 1000 permutations. These metrics were used to quantify ecological processes that influence microbial spatio‐temporal community structure and to distinguish and assess the relative contributions of deterministic and stochastic processes to community assembly within each sample using R package NST (v3.1.10) and iCAMP (v1.5.12) [102, 103]. Differential abundance analysis for ASVs was performed using package DESeq. 2 (v1.42.1) [104]. Potential microbial community functions were inferred using PICRUSt2 (v2.5.2) software [105]. Cohen's d effect sizes were estimated using the “cohen.d” function from R package effsize (v0.8.1). Guild‐level functional annotation of differential fungal ASVs was conducted based on the FUNGuild (v1.1) database [106].

### 5.5 Microbial network construction and analysis

Co‐occurrence network analysis was constructed by using the Spearman′s correlation method with Hmisc (v5.1‐3) and igraph (v2.0.3) packages, to identify potential microbial interactions relations and community structure among pairwise ASVs. ASVs with relative abundances less than 0.02%, prevalence less than 20%, and false discovery rate (FDR) greater than 0.05 were removed. Correlation thresholds were determined using the Random matrix theory (RMT) method computed by package RMThreshold (v1.1). All network properties were calculated using the subgraph function in igraph, and network visualization was done using the Cytoscape (v3.8.1) software [107]. R package microeco (v1.10.0) was employed to divide different modules from datasets by greedy modularity optimization method. The overall structural stability of the microbial networks was evaluated through robustness analysis, which generated metric characteristics of natural connectivity via several edge and node removal strategies. Two properties, within‐module connectivity (Zi) and among‐module connectivity (Pi), were calculated to determine the connectivity and identify the topological roles of each node [108]. Those nodes were classified into four subcategories: network hubs (Zi > 2.5 and Pi > 0.62), module hubs (Zi > 2.5 and Pi < 0.62), connectors (Zi < 2.5 and Pi > 0.62), and peripherals (Zi < 2.5 and Pi < 0.62). Phylogenetic tree of hub ASVs was constructed by MEGA11 based on Maximum Likelihood method with 1000 bootstrap replicates [109].

### 5.6 Mass spectrometry detection and metabolites analysis

For non‐targeted metabolomic profiling, methanol‐acetonitrile extracts from root and soil samples in the vegetative period were subjected to a liquid chromatography tandem‐mass spectrometry system composed of Waters Xevo G2‐XS QTOF high‐resolution mass spectrometer (Waters Corporation). In each data acquisition cycle, dual‐channel data acquisition can be performed on both low collision energy and high collision energy at the same time. The raw data files, collected via MassLynx (v4.2), underwent processing with Progenesis QI software for peak extraction and alignment. Metabolite identification was then performed against the online METLIN database, with the mass deviation for theoretical fragments maintained within 100 ppm. The identified compounds were functionally annotated and mapped to biological pathways using the Kyoto Encyclopedia of Genes and Genomes (KEGG), Human Metabolome Database, and LIPID metabolites and pathways strategy databases. OPLS‐DA were performed using the R package ropls (v1.40.0). The resulting model was validated with a 200‐repetition permutation test, and VIP scores were determined through multi‐fold cross‐validation. Differential metabolites were identified using a combined approach, requiring a specific VIP score from the OPLS‐DA model, and a fold‐change threshold from package limma (v3.64.3) [110]. Correlation analysis was performed to identify differential metabolites associated with the microbial community assembly process. The R package GSVA (v2.2.0) was used to calculate the gene set variation enrichment scores [111]. Furthermore, high‐performance liquid chromatography was used to quantify the contents of ferulic acid and ligustilide in RAS.

### 5.7 AMF colonization and abundance

The AMF colonization rate of RAS in harvest stage was determined using a previously described method with some modifications [112, 113]. Briefly, cleaned fine root segments (approximately 1 cm) of *A. sinensis* were immersed in potassium hydroxide (KOH, 10% w/v) solution at 90°C for 30 min, facilitating root tissue translucency. The roots were then thoroughly rinsed with distilled water until no residual alkali remained. Subsequently, the roots were acidified in a 5% glacial acetic acid (v/v) for 5 min at room temperature and rinsed again with distilled water. Staining was performed by immersing the roots in a blue–black ink solution (5% v/v, dissolved in glacial acetic acid) at 90°C for 3 min. After staining, the samples were decolorized in distilled water containing a few drops of glacial acetic acid for approximately 20 min. Finally, the processed root segments were mounted on glass slides to observe and photograph AM fungal colonization structures (e.g., hyphae, vesicles, and arbuscules of AM fungi) at 100/400× magnification using a Leica ICC50 W light microscope. Gridline intersection method was employed to determine the AMF colonization rate. The length of the hyphae was quantified with Fiji software [114]. Absolute AMF copy numbers were measured by quantitative real‐time polymerase chain reaction with the primer pairs AMV4‐5NF (5'‐AAGCTCGTAGTTGAATTTCG‐3') and AMDGR (5'‐CCCAACTATCCCTATTAATCAT‐3'). AM fungi 18S rRNA amplicon sequencing was performed as protocols described above by ITS, and the resulting sequences were taxonomically annotated against the MaarjAM database [115]. Besides, the rhizosphere soil samples at maturation stage were fractionated into four grades by dry sieving, including large macroaggregates (>2 mm in diameter), medium macroaggregates (0.9–2 mm), small macroaggregates (0.25–0.9 mm), and microaggregates (<0.25 mm). To evaluate the aggregation and stability of soil aggregates, we calculated the geometric mean diameter (GMD) index using the following formula: GMD = exp ($\sum_{i=1}^{n}\omega_{i}\;\ln\bar{x_{i}}\;$/ $\sum_{i=1}^{n}\omega_{i}$), where *n* represents the number of aggregate size fractions, $\bar{x_{i}}$ is the mean diameter of each size classes, *ω*_*i*_ is the aggregate weight retained in the *i*th sieve.

### 5.8 Proteomic profiling

Protein extraction was performed using RIPA‐based lysis (Thermo Fisher Scientific, Inc.) with homogenization and sonication. Following centrifugation, supernatants were reduced (DTT) and alkylated (IAM), acetone‐precipitated at low temperature, and reconstituted in NH_4_HCO_3_ (Sigma‐Aldrich). Proteins were digested with trypsin overnight at 37°C. Peptides were desalted, vacuum‐concentrated, reconstituted in 0.1% formic acid, and clarified by centrifugation prior to MS acquisition. Quantitative proteomic analysis was performed using a data‐independent acquisition (DIA) workflow based on a next‐generation Orbitra Astral high‐resolution mass spectrometer (Thermo Fisher Scientific, Inc.). Raw mass spectrometry (MS) files were processed and analyzed using the MaxQuant (v1.6.2.10) software by searching against target protein database. Proteins were assigned to orthologous groups and mapped to relevant biological pathways by querying the Clusters of Orthologous Groups of proteins and KEGG public databases, respectively. The R package clusterProfiler (v4.16.0) was employed to carry out KEGG enrichment analysis. Differentially expressed proteins were identified using limma and subsequently subjected to a homology search against the related species *Daucus carota* L. in Search Tool for Retrieval of Interacting Genes/Proteins (STRING, v12.0) database (https://string-db.org/) to facilitate PPI network analysis [116]. The intersection proteins with a high degree were regarded as hub proteins, and the PPI network was visualized based on Cytoscape. The R package linkET (v0.0.74) was used to perform Mantel tests between bioactive compounds, key metabolic pathways, and significantly enriched endophytic bacteria and AM fungi.

### 5.9 Transcriptomic profiling of a *Bacillus*‐*Arabidopsis* co‐culture system

*Arabidopsis thaliana* Col‐0 seeds were sterilized in 75% ethanol for 1 min, rinsed three times with sterile water, incubated in 2% sodium hypochlorite for 7 min, and then rinsed 4–5 times to fully remove the sterilant. Seeds were plated on MS agar, cold‐stratified at 4°C for 2–3 d and grown under controlled conditions (23 ± 2°C, 16 h light/8 h dark, 60% humidity). *Bacillus amyloliquefaciens* (BA) was first refreshed on LB agar. A single colony was grown overnight in LB broth at 37°C (180 rpm). Bacterial cells were collected, washed three times, resuspended in sterile liquid medium, and adjusted to 1.0 × 10^7 CFU/mL. Ten‐day‐old seedlings with similar growth were transferred into 24‐well plates and exposed to BA at 1.0 × 10^5 CFU/mL; controls received the same volume of sterile medium. After 3 d, roots were quickly separated from shoots using sterile tools. Shoots and roots were frozen immediately in liquid nitrogen and stored at –80°C. For each condition, 100 seedlings were pooled, with three independent biological replicates.

Total RNA was extracted from shoots and roots using the RNeasy Plant Mini Kit. RNA integrity and purity were assessed by 1.5% agarose gel electrophoresis and NanoDrop 2000C (OD_260_/_280_). Qualified RNA was used for cDNA library construction. Library quality was evaluated by RT‐PCR, and libraries with an effective concentration > 2 nM were sequenced on an Illumina HiSeq. 2500 platform (paired‐end). Clean reads were mapped to the *Arabidopsis* reference genome (TAIR10) for quantification. Differential expression was assessed using DESeq. 2 with thresholds of fold change > 1 and FDR < 0.05 (FDR obtained by multiple‐testing correction). KEGG enrichment analyses were performed using the R package clusterProfiler.

### 5.10 Statistical analysis

Group‐wise differential analyses, focusing on multiple treatment‐versus‐control comparisons, were conducted using a predefined statistical framework in R. The data distribution of each dataset, including phenotypic traits, microbial diversity indices from 16S rRNA and ITS sequencing, AMF‐related metrics, and root and soil nutrient contents, were first tested for normality using the Shapiro–Wilk test. Variance homogeneity among groups was examined using Levene's test from the package car (v3.1‐3). Data satisfying the assumptions of both normality and homoscedasticity were analyzed using Dunnett′s multiple comparison procedure (DescTools package, v0.99.60) to detect differences between each treatment and the control. In case of normal distribution but heterogeneous variances, the Games–Howell method was used (PMCMRplus package, v1.9.12). For datasets that were non‐normally distributed or had unequal sample sizes, a non‐parametric Dunnett's test (nparcomp package, v3.0) or Conover's post hoc (PMCMRplus) method was performed. Dunnett and Conover′s post‐hoc tests were corrected for multiple testing using the FDR approach, and the Games‐Howell test was performed with its default built‐in multiple‐comparison adjustment. All tests were two‐tailed, and statistical significance levels were indicated as *p* < 0.05 (*), *p* < 0.01 (**), and *p* < 0.001 (***). SEM was used to test hypothesized causal relationships among experimental treatments, microbe–soil–plant interaction, and plant performance. The SEM models were constructed based on prior biological hypotheses and literature evidence and were fitted using the package piecewiseSEM (v2.3.1) in R. We constructed linear models (LMs) to quantify the contribution of predictor variables, including RAS phenotype, AMF colonization, nutrient levels, and soil aggregates characteristics, to herb yield and bioactive constituents (response variable) under distinct inoculation treatments. The resulting relative effects across traits and treatments were visualized in a radar chart to highlight the contribution profile of each treatment.

## AI STATEMENT

The initial draft of this manuscript was partially polished for grammar and sentence structure using DeepSeek R1 (DeepSeek Inc.) and Gemini‐Exp‐1206 (Google Inc.). All AI‐generated contents were meticulously reviewed and edited by the authors to ensure factual accuracy and prevent any potential AI‐induced bias. We hereby confirm that no AI tools were employed in the processes of data generation, analyses, or visual content creation.

## AUTHOR CONTRIBUTIONS

**Zongyu Zhang**: Conceptualisation; investigation; methodology; data curation; validation; visualisation; software; writing—original draft preparation. **Yun Wang**: Methodology; writing—review and editing; **Jiecai Zhao**: Investigation; methodology; **Gaofeng Chen**: Investigation; project administration; data curation; **Andéole Niyongabo Turatsinze**: Investigation; writing—review and editing; **Liang Yue**: Investigation; methodology; validation. **Yuan Tian**: Methodology; validation. **Xiaofan Xie**: Investigation; visualization. **Ailing Ye**: Investigation; methodology. **Meilan Zhang**: Software; **Lingling Wu**: Methodology; visualization. **Zengxiang Guo**: Investigation; methodology; resources. **Yubao Zhang**: Resources; writing—review and editing. **Ruoyu Wang**: Funding acquisition; conceptualization; methodology; project administration; resources; supervision; writing—review and editing. All authors have read the final manuscript and approved it for publication.

## CONFLICT OF INTEREST STATEMENT

The authors declare no conflicts of interest.

## ETHICS STATEMENT

No animals or humans were involved in this study.

## Supporting information


**Figure S1.** Evaluation of the evolutionary relationships of candidate bacterial strains.
**Figure S2.** Dilution curve of bacterial 16S and fungal 18S gene sequence.
**Figure S3.** Alpha diversity response to bio‐inoculant treatments.
**Figure S4.** Principal Coordinates Analysis of the microbial community composition in the study samples.
**Figure S5.** The relative influence of different community assembly processes between inoculant‐treated and the control groups.
**Figure S6.** Relative abundance of the microbial community in the study samples.
**Figure S7.** Functional potentials of bacterial communities between CMA and CK soil samples.
**Figure S8.** Prediction of FUNGuild phenotype of fungi under different treatments.
**Figure S9.** Construction and validation of OPLS‐DA model.
**Figure S10.** Correlation between βNTI and relative abundance of the top differential metabolites.
**Figure S11.** Gene set variation analysis (GSVA) enrichment analysis.
**Figure S12.** Principal coordinate analysis and alpha diversity analysis in root endophytic bacterial community structures across CMA and CK samples of harvest stage.
**Figure S13.** KEGG pathway enrichment analysis of differentially expressed genes.
**Figure S14.** Distribution of rhizosphere soil and root nutrient profiles, including carbon, nitrate nitrogen, ammonium nitrogen, phosphorus, and potassium.

**Table S1.** Evaluation of multiple plant growth enhancement and disease resistance attributes of the PGPR strains.
**Table S2.** Effects of bacterial bioinoculants on yield, root coarse bark disease control, and herb quality of RAS.
**Table S3.** The number of order‐ and genus‐level ASVs showing statistically significant differences between treatment groups and control/CMA.
**Table S4.** Taxonomic affiliation of keystone microbial ASVs detected in root‐ and soil‐associated global co‐occurrence networks.
**Table S5.** Relative abundances of individual differential metabolites between different treatment and CK.
**Table S6.** Sequence alignment outcomes of differential proteins mapped against the related species *Daucus carota* L. for protein‐protein interaction (PPI) network construction.

## Data Availability

Raw amplicon sequence, metabolomic, and proteomic datasets obtained in the study have been deposited in the Genome Sequence Archive (CRA031456, https://ngdc.cncb.ac.cn/gsa/browse/CRA031456) and OMIX (OMIX012374, https://ngdc.cncb.ac.cn/omix/release/OMIX012374; OMIX012375, https://ngdc.cncb.ac.cn/omix/release/OMIX012375) at China National Center for Bioinformation/Beijing Institute of Genomics, Chinese Academy of Sciences under the BioProject numbers PRJCA047715 (https://ngdc.cncb.ac.cn/bioproject/browse/PRJCA047715). The raw transcriptome sequencing data have been deposited in the NCBI database (PRJNA756375) (https://www.ncbi.nlm.nih.gov/bioproject/PRJNA756375). The data and scripts used are saved in GitHub: https://github.com/zhangzy-bio/2025_Angelica-sinensis. Supplementary materials (figures, tables, graphical abstract, slides, videos, Chinese translated version, and update materials) may be found in the online DOI or iMetaOmics Science http://www.imeta.science/imetaomics.
