# Supplementary material for: Functionally complementary bacterial inoculant coordinates arbuscular mycorrhizal fungi to improve Angelica sinensis root yield and quality
Source: IMetaOmics. 2026 Mar 14;3(2):e70091. doi: 10.1002/imo2.70091 (PMC13674462; doi:10.1002/imo2.70091)
Supplement: Supplementary file 1 [file IMO2-3-e70091-s001.docx]

Supporting information to

**Functionally complementary bacterial inoculant coordinates arbuscular mycorrhizal fungi to improve *Angelica sinensis* root yield and quality**

**Running title:** Synergistic bacteria and fungi enhance *Angelica sinensis* yield and quality

Zongyu Zhang^1,2^, Yun Wang^1,3^, Jiecai Zhao^1^, Gaofeng Chen^1,4^, Andéole Niyongabo Turatsinze^1,5^, Liang Yue^1,2^, Yuan Tian^1,5^, Xiaofan Xie^1,5^, Ailing Ye^1,5^, Meilan Zhang^1,5^, Lingling Wu^1,5^, Zengxiang Guo^6^, Yubao Zhang^1,2^, and Ruoyu Wang^1,2*^

^1^State Key Laboratory of Ecological Safety and Sustainable Development in Arid Lands, Northwest Institute of Eco-Environment and Resources, Chinese Academy of Sciences, Lanzhou 730000, China

^2^Lanzhou Eco-Agriculture Experimental Research Station, Lanzhou 730000, China

^3^Key Laboratory of Desert and Desertification, Northwest Institute of Eco-Environment and Resources, Chinese Academy of Sciences, Lanzhou 730000, China

^4^Gansu Shangnong Biotechnology Co. Ltd, Baiyin 730900, China

^5^University of Chinese Academy of Sciences, Beijing 100049, China

^6^Gansu Minxian Angelica Research Institute, Dingxi 743000, China

*Correspondence: [wangruoyu@lzb.ac.cn](mailto:wangruoyu@lzb.ac.cn) (Ruoyu Wang)

**Figure S1 Evaluation of the evolutionary relationships of candidate bacterial strains.** Phylogenetic relationships (16S rRNA) of our previously isolated PGPR strains from diverse origins based on Maximum Likelihood method with 1000 bootstrap replicates and bootstrap values higher than 50% are shown on the nodes.

**Figure S2 Dilution curve of bacterial 16S and fungal 18S gene sequence.**

**Figure S3 Alpha diversity response to bio-inoculant treatments.** Effect of bio-inoculant treatments on bacterial and fungal alpha diversity indices (Chao1, Shannon, and Richness) in root and rhizosphere soil. Data are presented as mean ± SD. Asterisks indicate significant differences from the control (* *p* < 0.05, ** *p* < 0.01, and *** *p* < 0.001).

**Figure S4 Principal coordinate analysis of the microbial community composition in the study samples.** Principal coordinate analysis (PCoA) and permutational multivariate analysis of variance (PERMANOVA) depicting variation in bacterial and fungal community structures across root and rhizosphere soil samples in response to different treatments.

**Figure S5 The relative influence of different community assembly process between inoculant-treated and the control group.**

**Figure S6 Relative abundance of the microbial community in the study samples.** Phylum-, order-, and genus-level taxonomic distributions of bacterial and fungal communities from root and rhizosphere soil samples, illustrating changes in relative abundance under various microbial inoculant applications.

**Figure S7 Functional potentials of bacterial communities between CMA and CK soil samples.** (A) Bar charts show mean value with standard deviation (SD) for each category. Error bars indicate standard errors (SE), and each point represents the percentage change in effect size of bacterial community functional potential between CMA and CK, with 95% confidence intervals (CIs). Means below zero indicate higher functional potential in CK, whereas values above zero reflect greater potential in the treatment group. (B) Boxplots showing the ASV abundances of the top bacterial taxa associated with the key differential pathways in rhizosphere soil samples (Figure 2F). The x-axis lists bacterial taxa (two bars per taxon: CMA and control), and the y-axis indicates ASV abundance. Within each taxon, statistically significant differences between the control and treatment groups are marked with asterisks: * *p* < 0.05, ** *p* < 0.01, *** *p* < 0.001.

**Figure S8 Prediction of FUNGuild phenotype of fungi under different treatments.** FUNGuild-based functional annotation of differentially abundant fungal ASVs in root and rhizosphere soil samples across treatments. Colors within each bar indicate trophic mode categories and their relative proportions (%).

**Figure S9 Construction and validation of OPLS-DA model.** (A) OPLS-DA model validation based on root and rhizosphere soil compartments from different treatment groups. (B and C) Construction and validation of OPLS-DA statistical model of root and rhizosphere soil metabolites across CMA and CK samples.

**Figure S10 Correlation between βNTI and relative abundance of the top differential metabolites.** Scatter plots showing the correlation between βNTI and the relative abundance of the top differential metabolites detected from Figure 4B of rhizosphere soil samples. The top two panels show the relative abundance of key metabolites correlated with the microbial community assembly in CMA, whereas the bottom five panels depict those associated with CK. Grey-shaded area indicates the 95% confidence interval based on Pearson correlation analysis. Only statistically significant relationships (*p* < 0.05) are shown.

**Figure S11 Gene set variation analysis (GSVA) enrichment analysis.** GSVA of potential KEGG functions among all metabolites across different treatment groups in root and rhizosphere soil samples. The size of each point indicates the relative abundance of metabolites in the respective pathway. Plus signs (+) indicate that the pathway is significantly enriched in the treatment group relative to control, while minus signs (-) in the control group. Absence of any symbol indicates that the pathway abundance did not differ significantly between groups.

**Figure S12 Principal coordinate analysis and alpha diversity analysis in root endophytic bacterial community structures across CMA and CK samples of harvest stage.** (A) PCoA and PERMANOVA analysis. (B) Alpha diversity indices (Chao1, Shannon, and Richness) in root samples. Data are presented as mean ± SD. Asterisks indicate significant differences from the control (* *p* < 0.05, ** *p* < 0.01, and *** *p* < 0.001).

**Figure S13 KEGG pathway enrichment analysis of differentially expressed genes.** KEGG pathway enrichment analysis of differentially expressed genes (DEGs) in *Arabidopsis* shoots (A) and roots (B) following *Bacillus* inoculation.

**Figure S14 Distribution of rhizosphere soil and root nutrient profiles, including carbon, nitrate nitrogen, ammonium nitrogen, phosphorus, and potassium.** Data are presented as mean ± SD. Asterisks indicate significant differences from the control (* *p* < 0.05, ** *p* < 0.01, and *** *p* < 0.001).
